# Reproductive health seeking behaviour and its determinants in Indian women—a systematic review and meta-analysis

**DOI:** 10.3389/frph.2026.1769536

**Published:** 2026-03-25

**Authors:** Sananthya Karthikeyan, Saivarsaa Alwar, Rajiv Janardhanan, Madhumitha Haridoss

**Affiliations:** Division of Medical Research, SRM Medical College Hospital and Research Centre, Faculty of Medicine and Health Sciences, SRM Institute of Science and Technology, Kattankulathur, Chengalpattu, Tamil Nadu, India

**Keywords:** gynaecological morbidities, health-seeking, India, meta-analysis, reproductive disorders, reproductive health, systematic review, treatment-seeking

## Abstract

**Introduction:**

Reproductive health-seeking behaviour is critical to maternal health and overall well-being, yet many reproductive disorders remain underdiagnosed and contribute substantially to morbidity in India. Health-seeking for reproductive health conditions is often limited by symptom normalization, stigma, lack of awareness, and restricted access to female healthcare providers. This systematic review and meta-analysis aimed to quantify the prevalence of health-seeking behaviour for reproductive morbidities among Indian women and to summarise key factors influencing care utilisation.

**Methods:**

The protocol was registered in PROSPERO (CRD42024562508), and the review followed PRISMA 2020 guidelines. PubMed, Scopus, and Google Scholar were searched from inception to January 29, 2026, for studies reporting reproductive health-seeking behaviour among women in India. Eligible studies addressed reproductive morbidities, including menstrual disorders, gynecological infections, pregnancy complications, menopause-related problems, infertility, structural abnormalities, including uterine prolapse, pelvic organ prolapse, vaginal prolapse, and chronic pelvic pain. Data were extracted, risk of bias was assessed, and quantitative findings were synthesized using a meta-analysis of proportions with the inverse-variance method. Heterogeneity was examined using Cochran's Q and the I^2^ statistic.

**Results:**

The pooled prevalence of reproductive morbidities among women was 41.5% (95% CI: 31.2%–52.7%). Overall, 54.8% (95% CI: 46.0%–63.4%) sought treatment for at least one morbidity. Among care seekers, 31.4% used government facilities and 54.7% used private facilities. Allopathic medicine was the most common treatment modality (66%), followed by home remedies (27.9%), AYUSH therapies (12.7%), and over-the-counter medications (15.4%). While 47% perceived no need for treatment, the key barriers included symptom normalisation (60%), embarrassment (16.3%), lack of awareness (13.5%), financial constraints (12.3%), communication difficulties (27.3%), and distance to facilities (8.4%).

**Conclusion:**

This SRMA highlights substantial gaps in health-seeking among Indian women with reproductive morbidities, with only about half seeking formal treatment. Education and socioeconomic status remain key determinants of health-seeking, while limited awareness and poor knowledge of reproductive health services continue to impede timely treatment.

**Systematic Review Registration:**

PROSPERO CRD42024562508.

## 1 Introduction

Reproductive health-seeking behaviour among women is a critical public health issue with far-reaching implications for maternal and child health, family planning, and overall well-being. Understanding what influences women's decisions to seek reproductive healthcare, whether enabling or hindering, is essential for designing targeted, evidence-based interventions that improve access and outcomes (1). In many low- and middle-income countries, including India, reproductive health outcomes are closely linked to social norms, healthcare accessibility, and women's autonomy, making this an important area for research and policy focus.

Recent Indian data show that nearly one-third of women report at least one gynecological morbidity, such as abnormal vaginal discharge, menstrual problems, or pelvic pain, with notable increases over time in several states(NFHS-4 and NFHS-5) (2). Polycystic ovary syndrome (PCOS) has emerged as one of the most common endocrine and reproductive disorders, with estimates in Indian women of reproductive age ranging roughly from 9% to over 30%, suggesting that as many as one in five young women may be affected in some settings (3). Infertility remains another major concern: demographic and clinical studies from India indicate that up to about 8%–12% of couples may experience infertility, with primary infertility around 1.5%–2% and a higher burden of secondary infertility in certain regions. Pain- and menstrual cycle-related disorders, such as dysmenorrhea, are also highly prevalent among adolescents and young adults, frequently affecting more than half of participants in Indian studies and substantially impairing quality of life (4). Together, these conditions highlight that reproductive disorders constitute a significant and often under-recognized component of women's morbidity in India.

Despite this considerable burden, care-seeking for reproductive morbidities remains low. For instance, analysis of National Family Health Survey (NFHS-4) data indicates that only 39.2% of women with symptoms of reproductive morbidity sought treatment during 2015–16 (5). Multiple factors contribute to these low levels of healthcare utilization. Common barriers include the normalization of symptoms, limited access to female healthcare providers, long travel distances, and inadequate treatment availability (6). Gender dynamics further shape these behaviours, with women who have greater decision-making autonomy more likely to seek formal care (7). Socio-cultural norms also play a pivotal role, as reproductive and sexual health remain sensitive or taboo topics in many communities. Feelings of embarrassment, stigma, and lack of awareness often deter women from seeking appropriate care (8). Additionally, community-level factors such as education, socioeconomic status, and regional inequalities further contribute to disparities in access to reproductive healthcare (9).

Over the years, national surveys such as the NFHS, District Level Household and Facility Survey (DLHS), and Reproductive and Child Health Rapid Household Survey (RCH-RHS) have provided valuable data on fertility, maternal health, and healthcare utilization in India. However, findings remain fragmented across various health domains and populations. A systematic synthesis of the available evidence is therefore essential to understand the overall patterns and determinants of reproductive health-seeking behaviour among Indian women. This review aims to consolidate existing literature to identify key facilitators and barriers influencing healthcare utilization, providing a comprehensive evidence base to guide policies and interventions that enhance reproductive health outcomes for women in India.

## 2 Methods

### 2.1 Search strategy and selection criteria

The SRMA protocol was registered in PROSPERO (CRD42024562508) and conducted in accordance with the Preferred Reporting Items for Systematic Reviews and Meta-Analyses (PRISMA) 2020 guidelines. We conducted an advanced search utilizing the search strategies in PubMed, Scopus, and Google Scholar databases for studies on the health-seeking behavior of women's reproductive health in India, from its inception to January 29, 2026. The search terms were identified based on the PEOS approach, that is, P (Population): women at and above 15 years of age; E (Exposure):reproductive morbidities; O (Outcome): health-seeking behaviour and S (Setting): India. A strategy that maximizes sensitivity and precision was adopted to identify relevant studies. The detailed search strategy is presented in (Supplementary Tables S1–S3). Inclusion criteria comprise studies on women's reproductive health-related conditions, including menstrual disorders, gynecological infections, pregnancy complications, menopause problems, reproductive structural abnormalities include uterine prolapse, pelvic organ prolapase and vaginal prolapse, and chronic pelvic pain. Studies assessing reproductive health-seeking behaviors, such as seeking treatment and the type of healthcare facility visited, were included among women at and above 15 years of age. Studies focusing on urinary incontinence, HIV, cognitive impairment, neurodegenerative diseases, autoimmune diseases, and other disorders/diseases that do not exclusively affect reproductive health were excluded. The original protocol specified inclusion of benign conditions and malignancies affecting reproductive health. However, during full-text screening, we decided to exclude gynaecological cancers because the review focus was refined to non-malignant reproductive morbidities and their associated health-seeking behaviour. Cancer detection and treatment typically involve specialised oncology pathways, diagnostic work-ups, and referral systems distinct from routine reproductive care, which would have reduced clinical and methodological comparability across studies. This deviation from the protocol was agreed upon by the review team before data extraction and is reported here for transparency.

### 2.2 Study selection and data extraction

Two reviewers (ASV and SK) independently assessed the study's title and abstract (TiAb) against the eligibility criteria using Rayyan, a web-based tool. The full texts of the studies that passed the TiAb screening were reviewed. Any discrepancies in the decision (regarding inclusion or exclusion) were resolved by mutual consensus. A data extraction form was created in Microsoft Excel 365, and piloting of the data extraction process was conducted for five studies to standardize the Data Extraction Form (DEF). From the included studies, data were extracted independently by two reviewers (ASV & SK). The extracted data was verified for consistency and accuracy. Data on general study information, including author name, publication year, study title, study design, sample size, and location, were extracted. Study characteristics pertaining to the study population and outcome, including population demographics, symptom prevalence, treatment-seeking behavior, reasons for not seeking treatment, system of medicine, preferred health facility, barriers, influencing factors, and determinants of not seeking treatment, were also extracted. Studies utilizing secondary data, such as those based on the NFHS and other large-scale demographic or health surveys, were also considered eligible. These large scale suveys were categorized under the “survey type” subgroup to distinguish them from facility-based or community-based investigations as community-based secondary data. Studies were stratified by population groups such as tribal and general population. Place of residence is categorised as urban, rural, and both(urban and rural).

### 2.3 Risk of bias assessment

In the present review, all included studies were cross-sectional in design; therefore, the Appraisal Tool for Cross-Sectional Studies (AXIS) (10) was deemed appropriate and sufficient for assessing study quality. Although the protocol specified that cohort studies would be included, none met the eligibility criteria during full-text screening; therefore, the Newcastle–Ottawa Scale (NOS) was not applicable. The risk of bias assessment was conducted independently by two reviewers (ASV and SK) using the AXIS tool, which comprises 20 items with responses of “Yes,” “No,” or “Do not know.”

The tool evaluates eight key methodological domains: clarity of study objectives and appropriateness of study design; adequacy and justification of sample size; definition of the target population and representativeness of the sampling strategy; validity and reliability of measurement instruments; assessment of response rates and potential non-response bias; appropriateness of statistical methods; transparency in reporting results and acknowledgement of study limitations; and ethical considerations, including approval and informed consent. Together, these domains provide a comprehensive appraisal of methodological quality and potential sources of bias in cross-sectional studies.

### 2.4 Data synthesis

In the meta-analysis, the proportion was used as the effect measure for all outcomes, including those related to individuals who sought treatment for the disease condition, their preferred health facility and system of medicine, and the reasons for not seeking treatment. The inverse variance method was used to pool the proportions, and a random-effects model employing the DerSimonian–Laird estimator was used to account for between-study variability (11). Visual assessment of the forest plots, the Cochran-Q test (reported as a chi-square statistic with *k–1* degrees of freedom) and I-squared (I^2^) statistics were used to assess heterogeneity among the included studies. An I-square value greater than 25% or a Cochrane-Q score less than 0.1 was considered an indicator of heterogeneity between the included studies. The heterogeneity was further investigated by the subgroup analysis. Two-sided *p* < 0.05 was considered to be statistically significant, except for the heterogeneity test. Studies that did not report appropriate/complete quantitative data were synthesized narratively. Publication bias was assessed using the Funnel Plot or Egger's test. All statistical analyses, including the meta-analysis, were performed using R (version 4.5.1) in RStudio (version 4.2.3). The PROSPERO protocol originally specified the use of STATA v16 for quantitative analysis; however, the final analysis was conducted using R Studio which offered greater flexibility for meta-analysis of proportions and graphical outputs. Forest plots were generated in R, displaying events and sample sizes for each study, and were deemed appropriate for presenting the pooled proportions.

## 3 Results

### 3.1 Study selection

**Figure 1:**
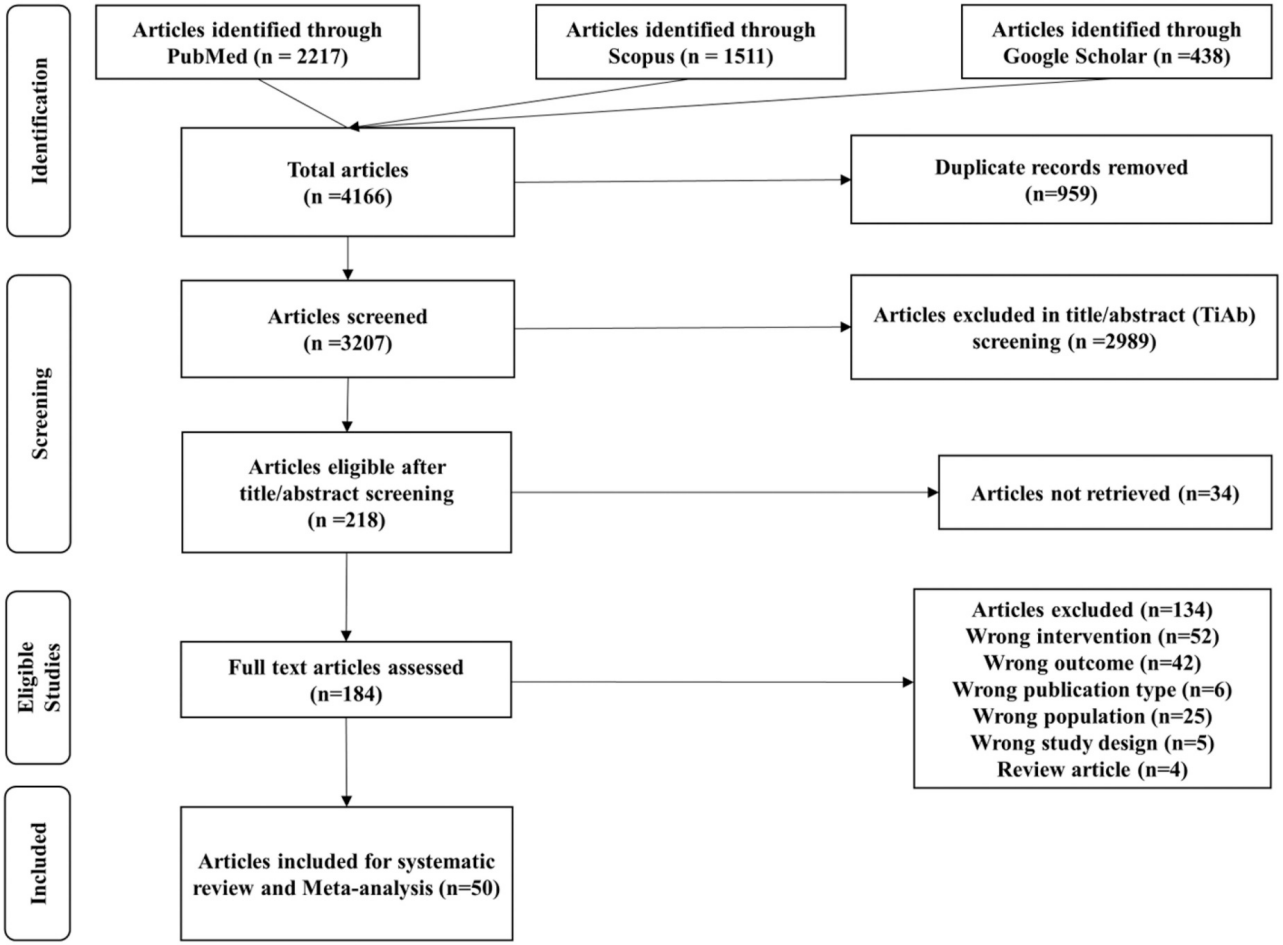
Study selection. PRISMA flowchart.

A systematic literature search was conducted in PubMed, Scopus, and Google Scholar, retrieving a total of 4166 studies. After removing 959 duplicates, 3,207 studies were screened based on title and abstract, of which 218 were deemed relevant for full-text retrieval. Following assessment against the inclusion criteria, 50 studies (2, 5, 6, 8, 12–57) were included, with quantitative data suitable for meta-analysis. The study selection process is illustrated in the PRISMA flowchart (Figure 1).

### 3.2 Characteristics of included studies

This review encompasses studies that assessed reproductive health-seeking behaviour among women at and above 15 years of age, ranging from adolescence to late adulthood. It provides a comprehensive overview of health-seeking patterns across reproductive disorders. The 50 studies included in the SRMA were assessed for a range of disease conditions, including Reproductive Tract Infections (RTIs) and related symptoms, menstrual problems, gynecological morbidities, postpartum morbidities, infertility, and Antenatal Care (ANC). Specifically, RTIs and related symptoms encompassed genital ulcers, abdominal pain, vaginal discharge, low backache, pain during urination, and spotting after sexual intercourse. Infertility-related conditions were assessed in terms of treatment-seeking behaviour and the utilization of full antenatal care services. Gynecological morbidities included vaginal bleeding, vaginal dryness, burning sensation, mood swings, foul-smelling vaginal discharge, excessive bleeding, and convulsions. Postpartum morbidities included high fever, severe headache, heavy bleeding, and lower abdominal pain. Menstrual problems included irregular menstruation, dysmenorrhea, fatigue, loss of appetite, vomiting, and cramps (Table 1).

**Table 1:** Characteristics of included studies.

S. No.	Study	Study Design	Study Setting	Study Population	Place of residence	Location	Disease condition	Sample size	Health facility utilization	Key findings
1	Bhilwar et al. 2015 (28)	Cross sectional study	Community	Tribal	Rural	Himachal Pradesh	RTIs and related symptoms	150	Not reported	In this study, 13.6% reported active RTI symptoms, but only 39.3% had heard of RTIs. 40% of women sought treatment, 40% sought advice from elderly women because of non availability of female doctors at the hospital.
2	Ravi and Kulasekaran 2013 (6)	Cross sectional study	Community	General Population	Rural	Thiruvarur district,Tamilnadu	RTIs	605	↑Government	In this community-based study, 14.5% reported at least one RTI symptom in the past six months, and just over half (52.3%) of those with symptoms sought treatment, all from public health facilities.
3	Patel. et al. 2023 (23)	Cross sectional study	Community	General Population	Rural	Bhopal,Madhya Pradesh	Mentural Problems	400	Not reported	In this study of adolescent girls, 27% reported menstrual problems, yet only about one-third of these sought treatment.
4	Goyal. et al. 2017 (31)	Cross sectional study	Community	General Population	Both	Allahabad	menopause	400	↑Private	In this study of postmenopausal women, with symptom (57% rural, 55% urban), treatment-seeking was markedly higher among urban women (71%) than rural women (29.5%), with urban women predominantly using private clinics.​
5	Sahu. et al. 2022 (24)	Cross sectional study	Community	General Population	Urban	Nanded city,Maharashtra	Gynecological Morbidities	750	↑Private	In this study of ever-married reproductive-age women, 75.7% reported at least one gynecological morbidity (most commonly menstrual irregularities at 46.8%, followed by reproductive tract infections at 25.2%), yet overall treatment-seeking was low at 33.1%, infertility showed the highest care-seeking (55.8%).
6	Sundhararajan. et al. 2023 (36)	Cross sectional study	Community	General Population	Urban	Kumbakonam,Tamilnadu	Peri Menopause	264	Not reported	In this cross-sectional study of peri- and post-menopausal school teachers (aged 40-60 years), 93.5% reported at least one menopausal symptom most commonly irritability (68.5%), joint/muscular pain (66%-74%), and physical/mental exhaustion (63–66%) yet only 31.8% sought health care services.
7	Shingade and Kazi 2015 (7)	Cross sectional study	Facility	General Population	Urban	Mumbai,Maharashtra	RTI/STI	273	↑Private	In this study of married women attending an STI/RTI clinic, only 47.6% had sought prior health care for their reproductive tract infection symptoms.
8	Pardeshi and Tambe 2017 (57)	Cross sectional study	Community	General Population	Urban	Pune,Maharashtra	Gynecological Morbidities	202	Not reported	In this study of ever-married women, 57% reported at least one gynecological morbidity, yet only 55% of symptomatic women sought treatment from formal providers. Treatment-seeking was significantly higher among women who discussed symptoms with their husbands.
9	Mathew and Francis 2017 (35)	Cross sectional study	Community	General Population	Rural	Udipi,Karnataka	Gynecological Morbidities	330	Not reported	In this cross-sectional survey of married women, 66.4% reported at least one gynecological symptom, of whom 63.9% had sought some form of treatment.
10	Abraham et al. 2014 (32)	Cross sectional study	Community	General Population	Rural	Thiruvanathapuram,Kerala	Gynecological Morbidities	540	↑Private	In this cross-sectional survey of women, 36.9% reported at least one gynecological morbidity. Only 55.3% of women with morbidity sought any treatment, with the highest care-seeking for infertility (94.1%) and lowest for menstrual problems (44.4%).
11	Hegde et al. 2013 (41)	Cross sectional study	Community	General Population	Urban	Bangalore,Karnataka	RTIs	179	↑Private	In this community survey, prevalence of RTI symptoms was 26.8% and 60% sought some form of treatment.
12	Singh et al. 2012 (13)	Cross sectional study	Community	General Population	Rural	India	Maternal Health care	3,599	Not reported	In this study, mothers who had live births in the prior 5 years, revealing low maternal care utilization: 14% full antenatal care, 46% safe delivery and 35% postnatal check within 42 days.
13	Varma et al. 2010 (56)	Cross sectional study	Community	Tribal	Rural	Visakhapatnam,Telengana	Antenatal Care	380	Not reported	In this mixed-methods study of mothers of children under 1 year across 24 villages, revealing high antenatal care (ANC) uptake exceeding India's national average: 90% tribal and 99% rural women received ≥1 check-up (mostly ≥3 visits),
14	Paul and Chellan 2007 (46)	Cross sectional study	Community based secondary data	General Population	Both	India	Post partum Morbidities	1,95,031	Not reported	In this study of women showed 20–30% treatment-seeking for reproductive health issues, low for menstrual/RTI.
15	Singh and Kumar 2014 (17)	Cross sectional study	Community based secondary data	General Population	Rural	India	Post partum Morbidities	1,74,913	↑Private	In this study, women who gave birth in the last 3 years found that 39.8% reported at least one postpartum morbidity in the first 6 weeks post-delivery. 55.1% of symptomatic women sought any treatment/consultation.
16	Ganganahalli and Singh 2022 (19)	Cross sectional study	Facility	General Population	Rural	Vijayapura,Karnataka	Antenatal Care	112	↑Private	In this study of rural postpartum women with 97% visiting private obstetricians for ANC, scans, investigations, or pregnancy-related complications.
17	Elizabeth et al. 2015 (40)	Cross sectional study	Community	General Population	Urban	Delhi	Reproductive Health care	253	Not reported	In this study, antenatal check-up and most knew recommended ANC components, TT and IFA. However, one-quarter had no ANC, nearly a quarter did not consume prescribed IFA. Postnatal treatment-seeking was particularly weak: 55.4% did not know how long rest is needed after delivery
18	Verma et al. 2019 (42)	Cross sectional study	Community	General Population	Rural	Uttarkhand	RTIs	450	Not reported	In this study, RTIs were investigated in 30 villages using a 30-cluster sampling technique, and 145 women were identified with RTIs, yielding a prevalence of 32.2%. Among these symptomatic women, only 14.5% reported receiving treatment, while 85.5% did not receive any care.
19	Tuddenham et al. 2010 (12)	Cross sectional study	Community	General Population	Rural	Murshidabad,West Bengal	Post partum Morbidities	2,114	Not reported	In this study, healthcare-seeking behavior for postpartum morbidities within 6 weeks of delivery was assessed. Overall, 5.8% of women did not seek any care, 49.2% consulted informal providers, and 45.0% sought care from formal health services.
20	Bhasin et al. 2020 (5)	Cross sectional study	Community based secondary data	General Population	Both	India	RTIs	91,818	↑Private	In this study, treatment-seeking for self-reported symptoms of reproductive tract infections (RTIs) among women were assessed. 11.3% reported RTI symptoms; only 39.2% of symptomatic women sought any advice or treatment.
21	Kumar et al. 2021 (16)	Cross sectional study	Community based secondary data	General Population	Urban	Uttar Pradesh and Bihar	Gynecological Morbidities	14,625	Not reported	In this study, 23.6% reported at least one gynaecological symptom, but only 31.8% of those sought treatment.
22	Sarkar et al. 2021 (26)	Cross sectional study	Community based secondary data	General Population	Urban	Kolkatta,West Bengal	Gynecological Morbidities	2,141	↑Private	In this study, 41.6% had never heard of these cancers and only 9.7% knew early symptoms; 80.5% first sought private care with mean 10-month symptom delay.
23	Surya et al. 2021 (53)	Cross sectional study	Community	General Population	Rural	Kancheepuram,Tamil Nadu	RTIs	330	Not reported	In this study, 50.3% reported at least one RTI symptom. Of those symptomatic, 60.8% sought some treatment, but home remedies were the most common option (43.6%), and only about one-third of all women reported seeking any formal care.
24	Sridharan et al. 2018 (25)	Cross sectional study	Community	General Population	Urban	Uttar Pradesh	Antenatal Care	5,666	Not reported	In this study, antenatal care (ANC) with utilization ranging from 23.7% among illiterate women without home ownership to 82.4% among literate women in the highest wealth group.
25	Kinkor et al. 2019 (20)	Cross sectional study	Community	General Population	Rural	Odisha	RTIs	3,567	Not reported	In this study, 47.1% sought treatment for RTI and related symptoms. Married women were significantly more likely, and unmarried adolescents less likely, to seek care.
26	Sabarwal and Santhya 2012 (49)	Cross sectional study	Community	General Population	Both	Andhra Pradesh, Bihar, Jharkhand, Maharashtra, Rajasthan and Tamil Nadu,	RTIs	2,742	Not reported	In this study, symptomatic young women, only about two-fifths of married and one-third of unmarried women sought care from a formal medical provider, with 57%.
27	Sharma et al. 2018 (37)	Cross sectional study	Community	General Population	Urban	India	RTIs	276	↑Government	In this study of married women of reproductive age, India, 35.5% reported RTI symptoms per WHO syndromic criteria. 57.1% of symptomatic women sought treatment.
28	Nair et al. 2012 (29)	Cross sectional study	Community	General Population	Urban	Thiruvananthapuram,Kerala	Mentural Problems	3,443	Not reported	In this study, 21.1% reported menstrual disorders but only 11.5% of those with menstrual problems had sought treatment, mainly from gynaecologists or general practitioners.
29	Pradahan et al. 2023 (22)	Longitudinal Ageing Study	Community based secondary data	General Population	Both	India	Gynecological Morbidities	18,547	Not reported	In this study 40% women showed reproductive healthcare-seeking.
30	Chellan 2004 (30)	Cross sectional study	Community based secondary data	General Population	Both	Tamilnadu	Gynecological Morbidities	18,040	Not reported	In this study, health-seeking behaviour for reproductive and sexual health problems among women in an Indian community setting. Treatment-seeking for gynecological morbidity is 15–35%.
31	Singh 2007 (39)	Cross sectional study	Community	General Population	Rural	Imphal,Manipur	RTI/STI	540	Not reported	In this study, reproductive tract infection related health-seeking behaviour was assessed. Formal healthcare utilisation remained suboptimal. Women frequently preferred self-care or traditional healers before approaching health facilities.
32	Arsude and Velhal 2023 (47)	Cross sectional study	Community	General Population	Rural	Ahmednagar	Antenatal Care	108	Not reported	In this study, health-seeking behaviour for reproductive health problems among women was suboptimal, with many women delaying or avoiding formal healthcare. Treatment-seeking was influenced by symptom severity.
33	Barua et al. 2024 (8)	Cross sectional study	Community	General Population	Urban	Uttar Pradesh	Reproductive Health care	317	Not reported	In this study, reproductive and gynecological morbidities among women reported low to moderate health-seeking behaviour.
34	Agarwal et al. 2022 (27)	Cross sectional study	Facility	General Population	Urban	Madhya Pradesh	RTI/STI	440	Not reported	In this study, treatment-seeking behaviour for RTIs/STIs was assessed. 71% of women had some knowledge of RTIs, this did not translate into practice. Treatment-seeking behaviour was significantly influenced by education, socioeconomic status, age, and menstrual hygiene practices.
35	Sarkar and Gupta 2015 (45)	Cross sectional study	Community based secondary data	General Population	Both	India	Infertility	6,43,944	Not reported	In this study, 80.6% of infertile women in India sought some form of treatment. However, only around half (49.9%) used allopathic care, while nearly 29% relied on non-allopathic or traditional treatments
36	Rani and Bonu. 2003 (43)	Cross sectional study	Community based secondary data	General Population	Both	India	Gynecological Morbidities	62,248	↑Private	In this study, found that a substantial proportion of women did not seek treatment despite experiencing symptoms, often normalising reproductive health problems. rural women treatment-seeking for reproductive issues 22%, urban 40%.
37	Girotra et al. 2023 (51)	Cross sectional study	Community based secondary data	General Population	Both	India	quality of ANC services	1,76,877	Not reported	In this study, health-seeking behaviour for reproductive and gynecological morbidities among women was inadequate, with a large proportion of symptomatic women not seeking timely medical care. Many women perceived symptoms as normal or transient and therefore delayed treatment.
38	Verma et al. 2015 (38)	Cross sectional study	Community	General Population	Both	Pooth Khurd and Darya Ganj, Delhi	treatment seeking behaviour for RTI symptoms.	215	Not reported	This study found that although reproductive health problems were commonly reported, treatment-seeking behaviour remained low to moderate. Women often relied on self-medication or informal advice before approaching health facilities.
39	Singh et al. 2016 (15)	Cross sectional study	Community based secondary data	General Population	Urban	India	Maternal Health care	15,415	Not reported	In this study, health-seeking behaviour for reproductive tract infections was suboptimal, with many women delaying care or seeking help from non-formal sources. Women with higher education and better access to health facilities were more likely to seek appropriate treatment.
40	Alcock et al. 2015 (14)	Cross sectional study	Community based secondary data	General Population	Urban	Mumbai	Maternal Health care	3,848	Not reported	In this study, women's decisions are shaped by education, wealth, migration status, and parity: poorer, less educated, high-parity and recent migrant women are less likely to seek adequate antenatal visits or facility delivery. As women gain resources and information, they increasingly choose private or tertiary public hospitals, bypassing local primary public facilities they perceive as low quality.
41	Adhikari et al. 2016 (18)	Cross sectional study	Community based secondary data	General Population	Urban	Chhattisgarh, Madhya Pradesh, Odisha and Rajasthan	Maternal Health care	14,058	Not reported	In this study, education showed the strongest association among women with 9th class or higher achieved 15–28% utilization vs. 2.6–11.4% among illiterate women.
42	Babu et al. 2016 (21)	Cross sectional study	Community	Tribal	Rural	Trivandrum	RTI	119	Not reported	In this study, 47% had gynecological morbidities yet health-seeking behavior was notably high at 77.3% despite 80% belonging to low socioeconomic status and 74.8% being anemic.
43	Patra and Unisa 2022 (55)	Cross sectional study	Community	General Population	Rural	Purab Medinipur and Dakshin Dinajpur of West Bengal	Infertility	159	Not reported	In this study, women who experienced infertility revealed complex treatment-seeking patterns where only 3 women sought no treatment, but treatment discontinuation was common. Health-seeking behavior was strongly influenced by socioeconomic factors.
44	Bala M et al. (54)	Cross sectional study	Community	General Population	Rural	Bisrakh block of district Gautam Budh Naga,Uttar Pradesh	ANC	172	Not reported	In this study, antenatal care utilization despite available government services. 9.9% received full antenatal care. 34.3% received no ANC check-ups.
45	Singh et al. 2022 (34)	Cross sectional study	Community	General Population	Urban	Faridabad,Haryana	RTI	149	Not reported	In this study, 46.3% had at least one RTI/STI symptom, yet only 28.9% sought treatment.
46	Shastri et al. 2015 (44)	Cross sectional study	Community	General Population	Rural	India	Reproductive health care	576	Not reported	In this study, 67% reported menstrual disorders, 56% had one or more reproductive problems and merely 18% consulted medical practitioners.
47	Kumar et al. 2016 (30)	Cross sectional study	Community	General Population	Urban	Chandigarh,India	Menstural problem	655	Not reported	In this study, prevalence of menstrual problems revealed 64.6%, yet only 25.3% sought treatment.
48	Kumar et al. 2019 (50)	Cross sectional study	Community based secondary data	General Population	Both	India	ANC	190898	Not reported	In this study, ANC utilization, only 21% received complete care ranging 2.3–65.9%.
49	Gupta and Raj 2024 (2)	Cross sectional study	NFHS	General Population	Both	India	Gynecological Morbidities	30433	↑Private	In this study, prevalence of gynecological morbidity ranged from 25.68% to 28.74%. Treatment-seeking remained from 35.05% to 38.16%.
50	Rangaraju et al. 2024 (52)	Cross sectional study	Community	General Population	Urban	India	Menopause	200	Not reported	In this study of women revealing poor quality of life in the physical domain. Health-seeking behavior showed 49% approached gynecologists, 54% visited family doctors, but 22.5% approached no one and 13% considered menopause “normal, not requiring medical attention,”

### 3.3 Risk of bias assessment for included studies

The study objectives were clearly stated across all 50 included studies. An appropriate study design was followed in 96% of studies, and 66% provided justification for their sample size. The sampling frame was drawn from the relevant population in 94% of studies, while 86% employed a selection process likely to yield participants representative of the target population. However, in 40% of studies, the measurement of risk factors and outcome variables was appropriate for the stated objectives. Statistical methods were described in sufficient detail to allow replication in 70% of the studies, and basic data were adequately reported in 86%. Measures to address and categorize non-responders were undertaken in 64% of studies. Internal consistency of results was observed in 96% of studies, and in 88% of studies, analyses were reported as described in the methods. In addition, 96% of studies presented discussions and conclusions that were supported by their findings. Finally, 72% of studies explicitly acknowledged their limitations. A detailed presentation of the AXIS quality assessment for all included studies is provided in graphical representation (Supplementary Figures S1–S4).

A total of 50 studies were included in this systematic review and meta-analysis. The included studies were published between 2003 and 29th January 2026, and the data were obtained from all states across India, including nationally representative surveys such as NFHS, DLHS, UDAYA, LASI, and RCH–RHS Rounds 1 and 2. The studies predominantly focused on the health-seeking behaviour of women experiencing reproductive morbidities. The distribution of studies across reproductive morbidity categories was as follows: 15 studies on RTIs and related symptoms, three studies on menstrual problems, eleven studies on gynaecological morbidities, three study on menopause, eleven studies on ANC, three studies on postpartum morbidities, two studies on reproductive healthcare utilisation, and two studies on infertility. Based on “survey type”, 31 studies were community-based, three studies were facility-based, and 12 studies utilized secondary data from community settings. Regarding study settings, three studies were conducted among tribal populations, and 43 studies were conducted among the general population.Place of residence 25 studies in rural areas, 22 studies in urban settings and five studies in both(urban and rural). Additionally, 13 studies reported reasons for not seeking healthcare for at least one symptom of reproductive morbidity (Table 1).

### 3.4 Prevalence of reproductive morbidities

A total of 50 studies were included in the systematic review and meta-analysis. Of these, 30 studies reported data on the prevalence of at least one symptom of reproductive morbidity among women in the included studies. The pooled prevalence of reproductive morbidities among women from included studies is 41.5% (95% CI: 31.2%–52.7%), with substantial heterogeneity across studies (I^2^ = 100%, *p* < 0.001) (Supplementary Figure S5). This indicates wide variability in prevalence estimates across the included studies, suggesting differences in study populations, settings, or methodologies. Subgroup analysis by disease condition revealed a prevalence of 34.5% for RTIs and related symptoms, 29.6% for menstrual problems, 46.5% for gynaecological morbidities, 38.2% for postpartum morbidities, 90.3% for menopause-related issues, 65.3% for reproductive healthcare, and 8.3% for infertility (Supplementary Figure S6). Despite stratification by disease category, heterogeneity remained high across all subgroups, suggesting that considerable variability persisted irrespective of condition type.

### 3.5 Treatment-seeking among those with reproductive morbidities

**Figure 2:**
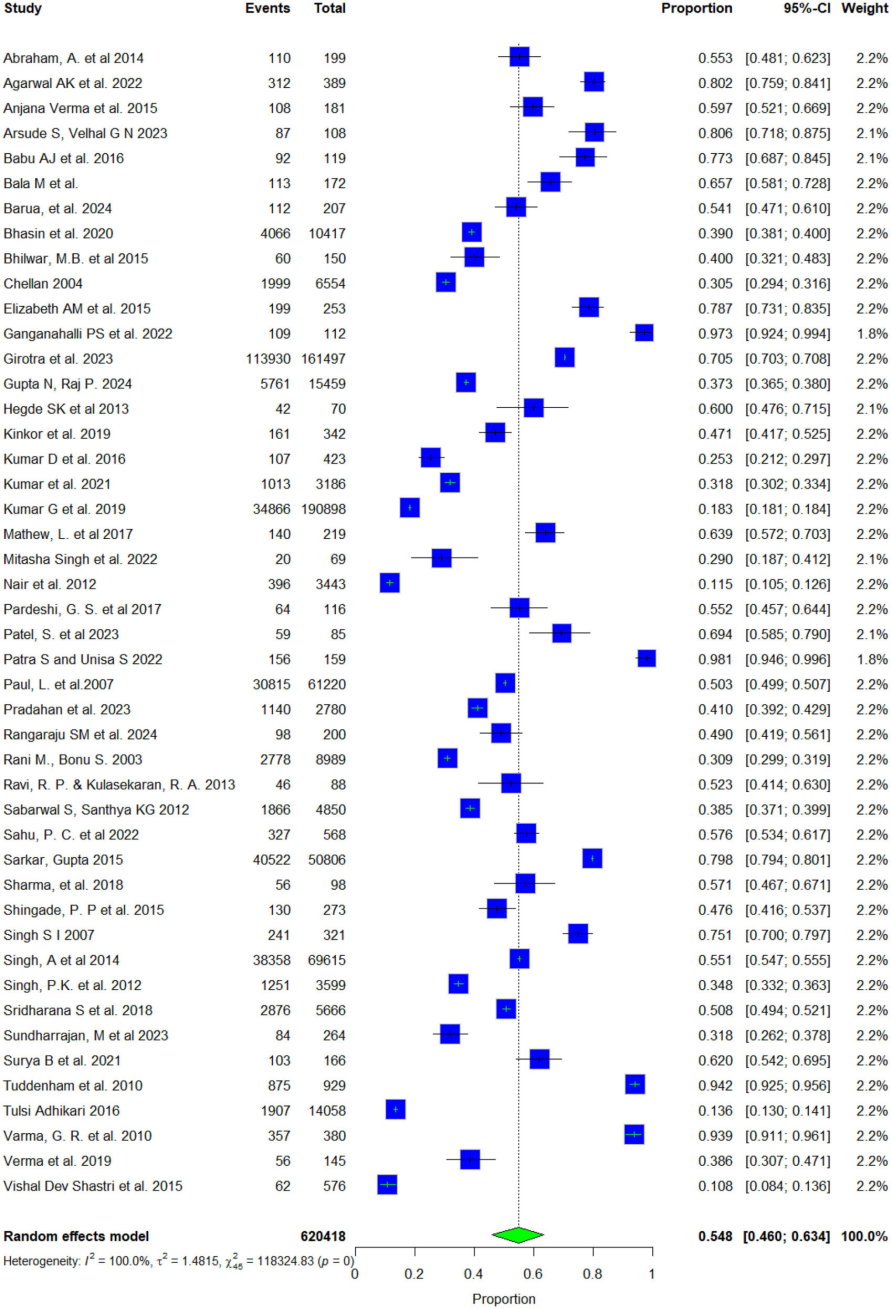
Forest plot showing pooled proportions of women who sought treatment for gynecological morbidities.

As the primary focus of this review, we assessed the proportion of women who sought treatment for their reproductive health conditions. Of the 50 included studies, 46 provided sufficient data for inclusion in the meta-analysis, which estimated the proportion of women who sought treatment for reproductive health conditions. The treatment-seeking proportion was calculated as the number of women who sought care divided by the total number of women with a reported reproductive morbidity. For studies in which all participants experienced reproductive morbidity, the total sample size was used as the denominator; in community-based studies, the reported prevalence was used. The pooled estimate indicated that 54.8% 95% CI: 46%–63.4%) of women sought treatment for reproductive morbidities, with extremely high heterogeneity across studies (I^2^ = 100%, *p* < 0.001) (Figure 2), Subgroup analysis by disease condition showed treatment-seeking proportions of 54.2% for RTIs and related symptoms, 63.5% for ANC, 31.4% for menstrual problems, 39.8% for gynaecological morbidities, 73% for postpartum morbidities, 40.1% for menopause-related issues, 67.5% for reproductive healthcare, and 93.1% for infertility (Supplementary Figure S7, Table S4). Despite stratification, heterogeneity remained high across all subgroups, indicating persistent variability between studies.

When stratified by population group (study setting), treatment-seeking patterns varied notably. The overall proportion of women seeking healthcare was 54.8%, with the highest proportion observed among tribal populations (76.6%, 95% CI: 35.4%–95.1%), followed by general populations (53.1%, 95% CI: 44.2%–61.8%). The pooled proportion of women sought healthcare by place of residence are rural (63.8%, 95% CI: 50%–75.6%), urban (47.2%, 95% CI: 37.3%–57.4%), and both (urban and rural) (40.7%, 95% CI: 34%, 47.8%) all showing significant heterogeneity (I^2^ = 100%) (Supplementary Figure S8, S9, Table S4).

Several of the included studies used secondary data sources, including the NFHS, DLHS, UDAYA, LASI, and RCH-RHS Rounds 1 and 2. These datasets provided population-level estimates that complemented findings from community-based studies. Treatment-seeking varied significantly by study setting: facility-based studies showed the highest rates (82.8%, 95% CI: 38.2%–97.4%), followed by community-based studies (57.5%, 95% CI: 46.8%–67.5%), and community studies based on secondary data (40.5%, 95% CI: 28.9%–53.3%), with substantial heterogeneity across all categories (I^2^ = 100%) (Supplementary Figure S10, Supplementary Table S4). Sensitivity analysis using the leave-one-out method revealed that the pooled proportion of women who sought treatment ranged from 0.53 to 0.56 when each study was sequentially omitted, indicating that the overall estimate was robust and not influenced by individual studies. However, heterogeneity remained materially unchanged, confirming that variability among studies persisted even after outlier exclusion (Supplementary Figure S11), suggesting considerable variability in treatment-seeking patterns across study designs, populations and settings. Publication bias was evaluated using funnel plots for the overall studies, which appeared symmetrical around their respective pooled effect estimates, suggesting a low likelihood of publication bias. Egger's regression tests confirmed no significant asymmetry overall (Bias estimate=−2.0087, t = −0.22, df = 44, *p* = 0.8268), with a *p*-value greater than 0.05, indicating that there is no statistically significant evidence of funnel plot asymmetry or publication bias among the included studies (Supplementary Figure S10).

**Figure 3:**
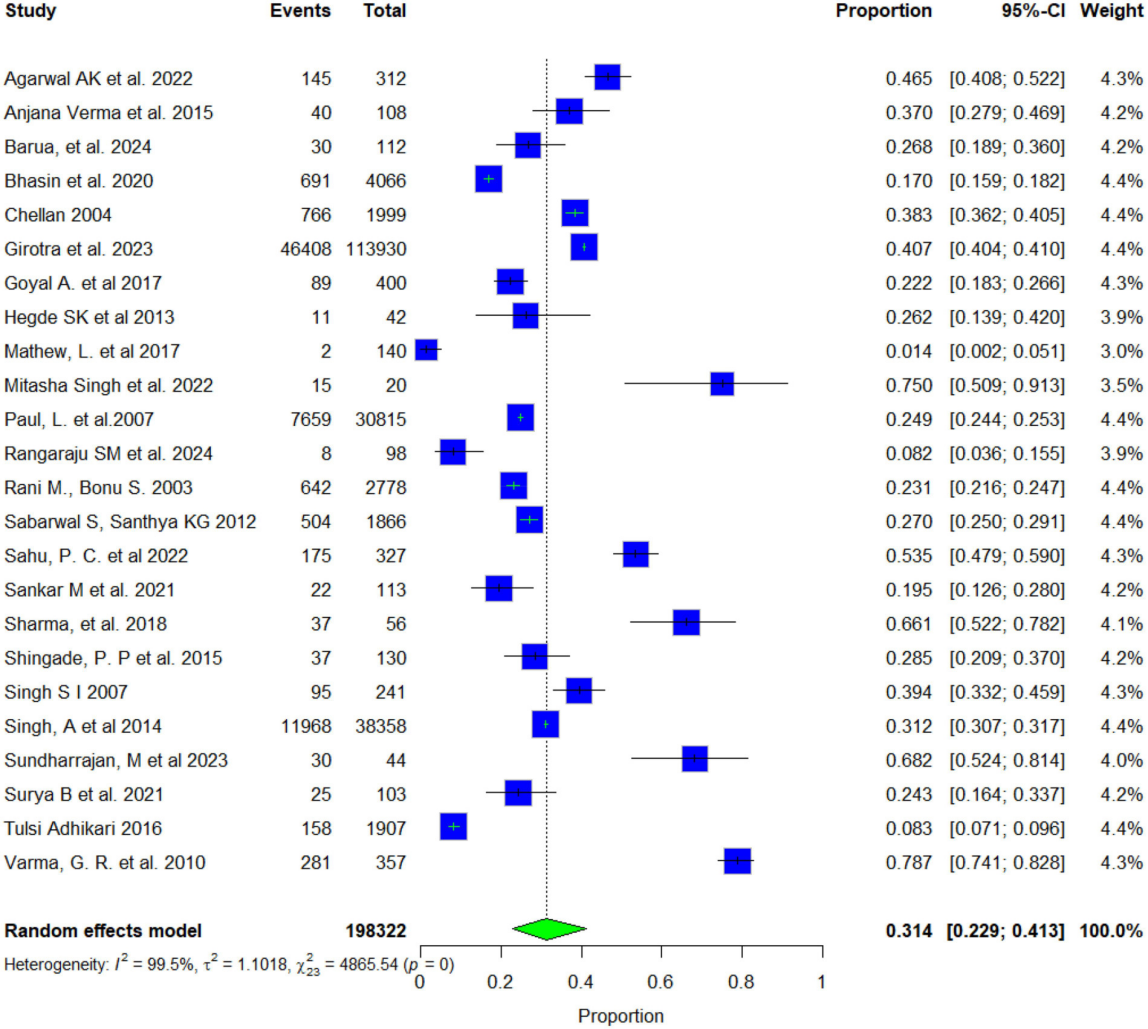
Forest plot of the pooled proportion of women who sought treatment from a public facility.

**Figure 4:**
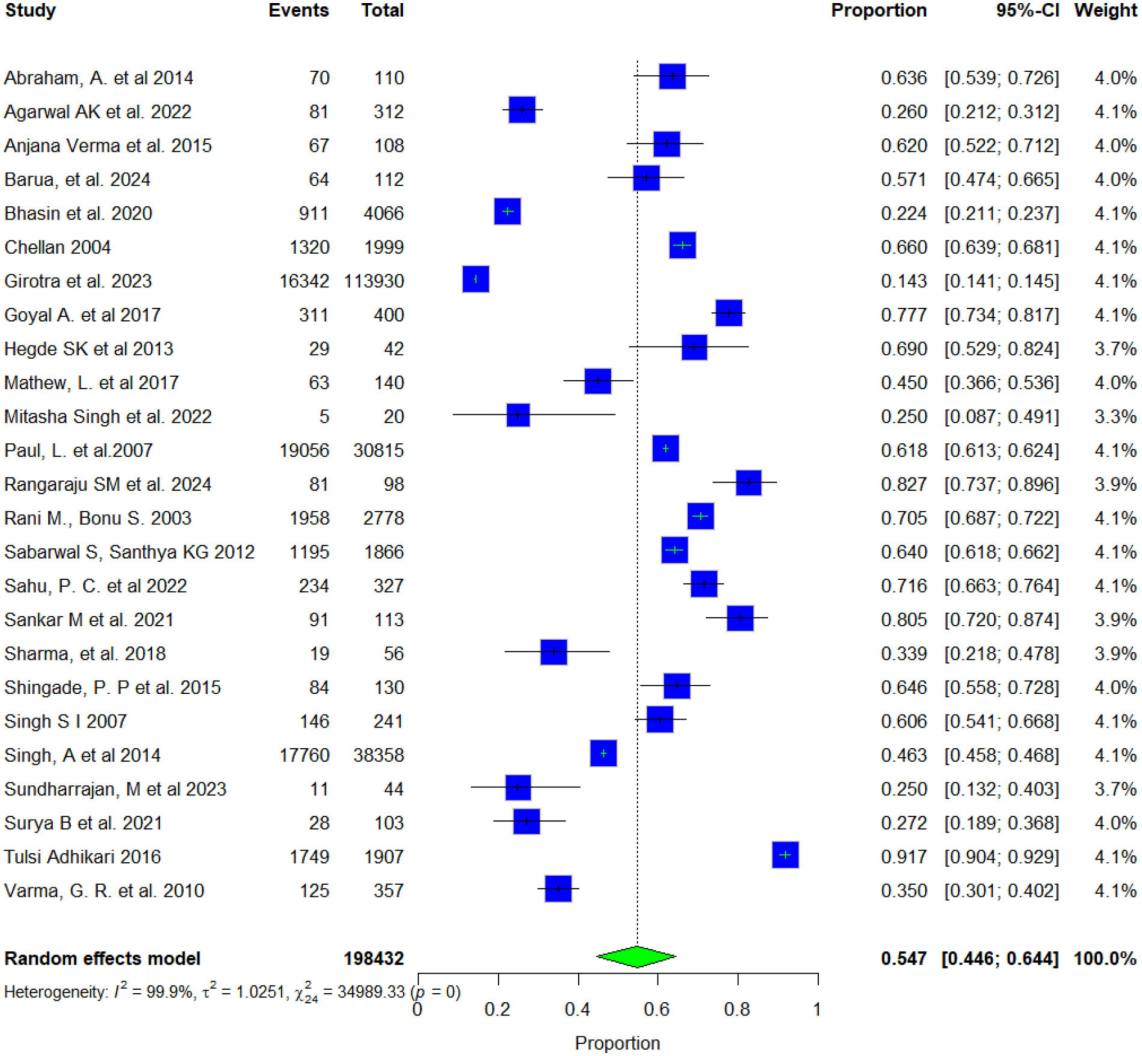
Forest plot of the pooled proportion of women who sought treatment from a private facility.

We also examined the types of health facilities women accessed for reproductive health concerns. Overall, 31.4% of women sought treatment from government facilities, while 54.7% used private facilities. The proportions varied notably across different reproductive health conditions (Figures 3, 4). Heterogeneity was extremely high for both government (I^2^ = 99.5%) and private (I^2^ = 99.9%) facility use, indicating substantial variability across studies. Sensitivity analyses revealed minimal changes in pooled estimates when individual studies were removed (government: 30.0%–33.7%; private: 52.3%–55.9%), confirming that the overall findings remained stable despite the marked heterogeneity (Supplementary Figures S13, S14). Publication bias was evaluated for government and private facilities using funnel plots, which appeared symmetrical around their respective pooled effect estimates, suggesting a low likelihood of publication bias. Egger's regression tests confirmed no significant asymmetry overall (Bias estimate = −4.2301, t = −1.25, df = 22, *p* = 0.2242) and (Bias estimate = 11.6399, t = 1.30, df = 23, *p* = 0.2228), with *p*-value is greater than 0.05, indicating that there is no statistically significant evidence of funnel plot asymmetry and no publication bias among the included studies (Supplementary Figures S15, S16).

Given India's diverse medical landscape encompassing allopathy alongside traditional systems such as Ayurveda, Yoga, Unani, Siddha, and Homeopathy, we examined the treatment modalities women used for reproductive morbidities. Our meta-analysis showed that 66% of women relied on allopathic medicine (95% CI: 42.3%–83.7%), while 16.2% used alternative medicine under the AYUSH systems (95% CI: 7.2%–32.5%), based on pooled estimates from six and seven studies, respectively. Both categories exhibited a high degree of heterogeneity (Supplementary Figures S17, S18). Use of home-based remedies such as herbal preparations, home-made decoctions, heat therapy, dietary changes, or other locally available ingredients was reported in six studies, with a pooled estimate of 27.9% (95% CI: 14.7%–46.5%) and substantial heterogeneity (I^2^ = 97.9%) (Supplementary Figure S19). Additionally, four studies reported the use of over-the-counter medicines, with a pooled proportion of 15.4% (95% CI: 11.8%–20%), also demonstrating notable heterogeneity (I^2^ = 55.7%) (Supplementary Figure S20).

### 3.6 Barriers to seeking treatment

As a substantial proportion of women did not seek treatment, the included studies further identified a range of barriers that hindered healthcare utilization. Overall, 9.1% of women did not seek care due to the high cost of treatment. 12.3% of women felt unable to undergo treatment due to financial constraints, 16.3% women felt shy to disclose their illness, and 60% women perceived the symptoms as normal rather than an illness. Family restrictions prevented 3.7% of women from seeking care, while 13.5% of women lacked awareness about available treatment options. Poor health was reported by 8.6% of women as a reason for not going to hospital to get treated, communication barriers with healthcare providers by 27.3% of women, and distance to the hospital by 8.4%. Additionally, 47% of women perceived no need for treatment, and 6% cited lack of time as a barrier (Supplementary Figures S21–S32). Determinants and influential factors related to reproductive health-seeking and healthcare utilization were synthesized qualitatively and summarized in a table (Supplementary Table S5).

## 4 Discussion

This systematic review and meta-analysis synthesized evidence from 50 studies that assessed reproductive health-seeking behaviour among Indian women at and above 15 years of age. The pooled prevalence of reproductive morbidities reported in the included studies is 41.5% (95% CI: 31.2%–52.7%), indicating that reproductive morbidities remain common among Indian women. Among women reporting any form of reproductive morbidity, only about 54.8% (95% CI: 46%–63.4%) sought treatment. The high heterogeneity (I^2^ = 100%) indicates that health-seeking patterns vary widely across populations, study settings, study designs, and health conditions.

Subgroup analyses provided a more nuanced picture. Treatment-seeking was highest for infertility (93.1%), compared to other reproductive morbidities. Notably, exceeding the global pooled 60% (range 25%–98%) influenced by education, income, age, and insurance (58). Menstrual disorders showed lower healthcare seeking, similar to menopause issues, driven by symptom recognition, perceived severity, and social acceptability. RTI/STI delays stemmed from poor symptom knowledge and superstitions; strengthening frontline worker counseling, repeated education, and patient-friendly clinics is essential (59).

Based on our observations, approximately 42.6% of the women did not seek any treatment for their reproductive morbidities (Supplementary Figure S33). This reflects the persistent barriers in availing reproductive healthcare services in India. The most common reasons reported for not seeking care were normalization of symptoms, embarrassment/shyness, lack of awareness, financial constraints, and distance to health facilities. Findings from this review indicate that women with better education, sound economic status, and the capacity to make decisions at the household level are much more likely to report seeking timely care for reproductive health issues. This finding is consistent with evidence from across South Asia, where women's empowerment is strongly linked to improved maternal and reproductive health outcomes, particularly where male-dominated decision-making delays or prevents care-seeking (60–62).

The type of health facility accessed is also an important indicator of women's health-seeking preferences. Results show that as many as 54.7% of the women sought care from private facilities compared to 31.4% seeking services from government facilities. Preference for private providers was especially high for gynaecological and postpartum morbidities, while government facilities were more often used for RTIs and antenatal care. Strengthening public reproductive health services, ensuring the availability of female providers, and building trust through quality care are key implications of these findings.

Disparities in treatment-seeking were also found across studies conducted in different settingsby population group are the tribal population (76.6%) and the general population (54.8%). By place of residence as rural (63.8%), followed by urban (47.2%) and both (urban and rural) (40.7%). This may suggest the effectiveness of targeted outreach and community health worker programs in certain tribal and rural areas, although data heterogeneity remains high (63). Facility-based studies reported higher treatment-seeking estimates (82.8%) compared to community-based surveys (57.5%), and community- based secondary surveys (40.5%) which is expected given the potential reporting and selection biases. Patterns of treatment modality offer additional insight into women's health-seeking behavior. The implication is that culturally sensitive education strategies are needed to promote timely and appropriate treatment while respecting traditional and cultural practices. mHealth interventions can enhance awareness and adherence to recommended maternal health practices. They can also serve as an educational platform to help women move beyond traditional beliefs and adopt modern maternal healthcare practices (64).

The factors identified in this review as barriers to reproductive health-seeking are similar to those reported in previous literature. Feelings of embarrassment, stigma, and social taboos often prevented women from seeking care, while the misconception that symptoms of RTIs were normal or self-limiting lowered perceived needs for treatment. A lack of awareness about partner treatment and available services further led to delays in care. Low education, socio-economic status, early marriage, and rural residence linked to high morbidity but low utilization, while health information exposure promoted use. Recent reviews highlight persistent issues like distrust in public facilities and weak ASHA/ANM outreach. Strengthening community-based programs and primary healthcare services may help bridge these gaps (65).

The findings highlight key priorities that enhance awareness through community and school-based reproductive health education, increase female healthcare providers to overcome gendered barriers, particularly in rural/tribal areas; strengthen public health systems for affordable, accessible services; and integrate education into programs like NFHS, RCH, and RMNCH + A. Community Advisory Boards (CABs) in LMICs like India can foster culturally sensitive engagement and equitable access. Prioritizing women's empowerment for decision-making and economic independence is foundational (66).

This review presents one of the most comprehensive syntheses to date on reproductive health-seeking behaviour among Indian women, integrating data from diverse populations and domains of reproductive health. However, several limitations should be acknowledged. Given the very high heterogeneity, the pooled estimates should be interpreted with caution. The meta-analysis was conducted to summarize overarching patterns; however, the variability limits the precision and comparability of the findings. Publication bias was low; however, reporting bias within primary studies could not be ruled out. Although multiple subgroup analyses were performed to explore sources of heterogeneity, very high between-study variability remained unexplained, likely reflecting differences in case definitions, measurement methods, study populations, and contextual factors across studies. Moreover, most included studies had a cross-sectional design, which limits causal inference between determinants and health-seeking outcomes. An additional limitation is that the pooled prevalence estimates of reproductive morbidities were derived exclusively from studies reporting reproductive health-seeking behaviour, which may not fully capture the true population burden of these conditions.

## 5 Conclusion

This systematic review and meta-analysis show that about half of Indian women with reproductive morbidities seek treatment, highlighting persistent challenges in access and healthcare utilization. Treatment-seeking behaviour is shaped by a complex interplay of educational, economic, gender, cultural, and structural factors within the health system. Strengthening reproductive health in India will require improving the quality and reach of public healthcare services, enhancing women's autonomy, and addressing cultural taboos through targeted health literacy and community engagement. Future research should adopt longitudinal and mixed-methods designs to better capture behavioural change over time and evaluate interventions aimed at improving reproductive health-seeking behaviour.

## Data Availability

The original contributions presented in the study are included in the article/Supplementary Material, further inquiries can be directed to the corresponding author.
