# Supplementary material for: Reproductive health seeking behaviour and its determinants in Indian women—a systematic review and meta-analysis
Source: Front Reprod Health. 2026 Mar 25;8:1769536. doi: 10.3389/frph.2026.1769536 (PMC13056874; doi:10.3389/frph.2026.1769536)
Supplement: Supplementary file 1 [file Table1.docx]

**Supplementary Material**

**Title:** **Reproductive health seeking behaviour and its determinants in Indian women- A systematic review and meta-analysis**

**Authors:** Sananthya Karthikeyan^1^, Sai varsaa Alwar, Rajiv Janardhanan^1^*^#^*, and [Madhumitha](https://pubmed.ncbi.nlm.nih.gov/?term=Haridoss+M&cauthor_id=38581635)

[Haridoss](https://pubmed.ncbi.nlm.nih.gov/?term=Haridoss+M&cauthor_id=38581635)^1#^

**Affiliation:**

^1^Division of Medical Research, SRM Medical College Hospital and Research Centre, Faculty of Medicine and Health Sciences, SRM Institute of Science and Technology, Kattankulathur, Chengalpattu, Tamil Nadu, India

| **Search #** | **Search words** | **Search hits as of 27-06-2024** |
| --- | --- | --- |
| E- Reproductive  health issue | Menstrual OR (Polycystic ovary  syndrome) OR Adenomyosis OR Endometriosis OR (Ovarian cysts) OR  leiomyomas OR (Vaginal atrophy) OR Menopause OR menopausal OR (Interstitial  cystitis) OR (Uterine prolapse) OR (Vaginal prolapse) OR Rectocele OR  Cystocele OR (Mullerian Duct Anomalies) OR (Uterine Didelphys) OR  (Bicornuate Uterus) OR (Septate Uterus) OR (Unicornuate Uterus) OR  Hypoplasia OR (Androgen Insensitivity Syndrome) OR (Congenital Adrenal  Hyperplasia) OR (Cloacal Anomalies) OR (Persistent Mullerian Duct Syndrome)  OR (Cervical cancer) OR (Ovarian cancer) OR (Endometrial cancer) OR  (uterine cancer) OR (Vaginal cancer) OR (vulvar cancer) OR (Menstrual  Disorders) OR Dysmenorrhea OR Menorrhagia OR Amenorrhea OR (menstruation) OR (Premenstrual)  OR PMS OR Gynecolog* OR (Bacterial Vaginosis) OR Candidiasis OR  Trichomoniasis OR (reproductive tract infection) OR RTI OR Chlamydia OR  Gonorrhea OR (Pelvic Inflammatory Disease) OR (Human Papillomavirus) OR  (Genital Herpes) OR Vulvitis OR Vaginitis OR (uterine cervicitis) OR  "pregnancy complications"[MeSH Terms] OR pregnancy OR  ("Infertility, Female"[Mesh]) OR infertil* OR antenatal OR Reproduc* OR Gynaecolog* OR maternal OR  Perinatal OR postpartum | 3908018 |
| O - Health Seeking  Behaviour | "Patient  Acceptance of Health Care"[Mesh] OR Health-seeking OR help-seeking OR  (health AND seek) OR (help AND seek) OR (health AND seeking) OR (help AND  seek) OR (help AND seeking) OR (treatment AND seeking) OR "treatment  seeking" OR care-seeking OR "care seeking" OR "Health  care utilization" OR "sought treatment" OR "sought  care" OR "sought help" | 322261 |
| S - India | India OR  "India"[Mesh] OR Indian | 985785 |
| E&O |  | 48447 |
| E&O&S |  | 1931 |

**Supplementary Table 1: Search strategy and results in Pubmed**

| **Search #** | **Search words** | **Search hits as of 27-06-2024** |
| --- | --- | --- |
| with all of the words | Seek India |  |
| with at least one of the words | Menstrual Ovary Ovaries Menopause  menopausal Cervical Ovarian Endometrial uterine Vaginal vulvar Gynecological  antenatal reproductive gynaecological maternal perinatal postpartum |  |
| without the words |  |  |
| where my words occur | in the title of the article | 435 |

**Supplementary Table 2: Search strategy and results in Google Scholar**

| **Search #** | **Search words** | **Search hits as of 27-06-2024** |
| --- | --- | --- |
| E- Reproductive  health issue | TITLE-ABS-KEY (  menstrual OR "polycystic ovary syndrome" OR adenomyosis OR  endometriosis OR "ovarian cysts" OR leiomyomas OR "vaginal  atrophy" OR menopause OR menopausal OR "interstitial  cystitis" OR "uterine prolapse" OR "vaginal prolapse"  OR rectocele OR cystocele OR "mullerian duct anomalies" OR  "uterine didelphys" OR "bicornuate uterus" OR  "septate uterus" OR "unicornuate uterus" OR hypoplasia  OR "androgen insensitivity syndrome" OR "congenital adrenal  hyperplasia" OR "cloacal anomalies" OR "persistent  mullerian duct syndrome" OR "cervical cancer" OR  "ovarian cancer" OR "endometrial cancer" OR  "uterine cancer" OR "vaginal cancer" OR "vulvar  cancer" OR "menstrual disorders" OR dysmenorrhea OR  menorrhagia OR amenorrhea OR "menstruation" OR  "premenstrual" OR pms OR gynecolog* OR "bacterial  vaginosis" OR candidiasis OR trichomoniasis OR "reproductive tract  infection" OR rti OR chlamydia OR gonorrhea OR "pelvic  inflammatory disease" OR "human papillomavirus" OR  "genital herpes" OR vulvitis OR vaginitis OR "uterine  cervicitis" OR "pregnancy complications" OR pregnancy OR  "infertility, female" OR infertil* OR antenatal OR reproduc* OR  gynaecolog* OR maternal OR perinatal OR postpartum ) | 4174647 |
| O - Health Seeking  Behaviour | TITLE-ABS-KEY  ( health-seeking OR help-seeking OR ( health AND seek ) OR ( help AND seek )  OR ( health AND seeking ) OR ( help AND seek ) OR ( help AND seeking ) OR (  treatment AND seeking ) OR "treatment seeking" OR care-seeking OR  "care seeking" OR "health care utilization" OR  "sought treatment" OR "sought care" OR "sought  help" ) | 269093 |
| S - India | TITLE-ABS-KEY(India OR Indian ) | 879720 |
| E&O&S |  | 1335 |

**Supplementary Table 3: Search strategy and results in Scopus**

| **Parameter** | **Number of studies for meta- analysis** | **Pooled estimate (Confidence Interval)** | **Heterogeneity** | | | | **Subgroup differences** | |
| --- | --- | --- | --- | --- | --- | --- | --- | --- |
|  |  |  | **I square (%)** | **Tau square** | **Chi square (Q) overall** | **p value** | **Chi square (Q)** | **p value** |
| **Disease Condition** |  |  |  |  |  |  |  |  |
| RTIs and related symptoms | 15 | 54.2% (45.6%-62.5%) | 97.2 | 0.4356 | 495.77 | <0.0001 |  |  |
| Menstural problems | 3 | 31.4% (8.2%-70.2%) | 98.9 | 2.0742 | 184.28 | <0.0001 |  |  |
| Gyneacological Morbidities | 9 | 40.1% (28%-53.5%) | 98.5 | 0.6777 | 518.29 | <0.0001 |  |  |
| Menopause | 1 | 31.8% (26.2%-37.8%) |  |  |  |  |  |  |
| ANC | 9 | 63.5% (35.2%-84.8%) | 100 | 3.132 | 91550.12 | 0 |  |  |
| Postpartum Morbidities | 3 | 73% (32.1%-94%) | 99.7 | 2.3769 | 658.79 | <0.0001 |  |  |
| Reproductive healthcare | 2 | 67.6% (40.5%-86.4%) | 96.7 | 0.628 | 30.21 | <0.0001 |  |  |
| Infertility | 2 | 93.1% (51.8%-99.4%) | 94.9 | 3.1582 | 19.59 | <0.0001 |  |  |
| overall |  |  |  |  |  |  | 32.08 | <0.0001 |
| **Survey Type** |  |  |  |  |  |  |  |  |
| Community | 30 | 57.8% (46.7% - 68.1%) | 99 | 1.513 | 2897.27 | 0 |  |  |
| Facility | 3 | 82.3% (38.9%-97.1%) | 98 | 2.9807 | 98.43 | <0.0001 |  |  |
| Community based secondary data | 11 | 40.8% (28.2%-54.8%) | 100 | 0.908 | 114241.3 | 0 |  |  |
| overall | 44 | 55.4% (46.2%-64.3%) | 100 | 1.5379 | 117593.94 | 0 | 5.59 | 0.0612 |
| **Study Setting** |  |  |  |  |  |  |  |  |
| Tribal | 3 | 76.6% (35.4%-95.1%) | 98.5 | 2.4452 | 136.9 | <0.0001 |  |  |
| Rural | 22 | 62.2% (47.6%-74.9%) | 100 | 1.9909 | 99914.88 | 0 |  |  |
| Urban | 18 | 46.5% (35.3%-58.1%) | 99.9 | 0.9996 | 18048.26 | 0 |  |  |
| General | 6 | 45.7% (30.5%-61.8%) | 99.6 | 0.659 | 1289.76 | <0.0001 |  |  |
| overall |  |  |  |  |  |  | 4.79 | 0.1875 |

**Supplementary Table 4: The pooled proportions of women who sought treatment for reproductive morbidities based on subgroup by Disease condition, Survey type and Study setting**

| **Determinant** | **No. of studies (n)** | **Direction of association with appropriate health‑seeking** |
| --- | --- | --- |
| Age (younger vs older women) | 4 | Women >35 years were significantly less likely to receive allopathic treatment compared with women <30 years. Several RTI studies observed higher treatment‑seeking among women aged 25–35 years compared with younger or older women (1)(2), and currently married women had higher RTI risk and treatment‑seeking than unmarried women(3)(4). |
| Education level | 10 | Completion of school education strongly reduced the risk of primary infertility and was associated with higher odds of seeking infertility treatment. ​ When both partners had completed schooling, odds of treatment‑seeking were substantially higher. ​ Women’s education improved awareness of ANC/PNC components and recognition of complications, and was repeatedly identified as an important determinant of utilisation(5)(6).​ RTI/gynaecological studies reported greater symptom recognition and care‑seeking among women with at least middle‑school education (7) (8)(9)(10)(11). |
| Marital status | 5 | Marriage at ≥30 years was associated with higher prevalence of primary infertility. Women married at ≥18 years were more likely to seek treatment than those married earlier. ​ RTI studies showed higher symptom burden and care‑seeking among currently married women(3)(4)(9). |
| Employment / work status | 3 | Unemployed women were less likely to seek treatment than those in professional/service/production jobs. RTI studies reported that women engaged in paid work were more likely to seek facility‑based care, even though work obligations sometimes limited time to attend clinics(12)(2). |
| Wealth (household / individual) | 4 | Higher wealth reduced the risk of primary infertility. ​ Women from richer households were more likely to use allopathic and private facilities for gynaecological/RTI symptoms than poorer women(7)(2)(5). |
| Affordability / income | 8 | Inability to afford allopathic services was a direct reason for not consulting formal providers. Low income constrained ANC/PNC and other reproductive health service use among slum women. Even where many participants were lower‑middle class or above, a subset still cited financial constraints as a major barrier(10). Cost or wage loss appeared repeatedly in RTI and gynaecological studies (13)(8)(14)(1)(15)(2). |
| Household wealth / MPCE / assets | 3 | Economically weaker women, defined by lower MPCE or asset quintiles, were significantly less likely to seek consultation or treatment than richer women. ​ Similar gradients were reported in ANC and RTI work where higher asset scores predicted greater service use(5)(6). |
| Caste / social group | 5 | Strong associations were observed between caste, socioeconomic inequalities and use of reproductive health services, with disadvantaged groups generally having lower utilisation. Scheduled Tribe women were least likely to seek treatment; SC/OBC/forward‑caste women had higher odds of seeking care. RTI/gynaecological studies also reported lower treatment‑seeking among ST/SC women compared with OBC/other castes(16)(4)(8). |
| Religion / region | 4 | Women in the northeast were about 50% less likely to seek treatment or consultation than women in central India, even after adjustment.​ Other studies described religious and regional patterns in provider choice and sector of use, including lower public‑sector ANC use and distinct provider preferences among Muslim women(6)(17)(4). |
| Place of residence (rural/urban/tribal) | 6 | Rural residence was associated with slightly higher infertility prevalence and lower or delayed use of formal services. Multiple RTI and gynaecological morbidity studies reported poorer care‑seeking in rural and tribal populations than in urban areas, with greater reliance on home remedies, informal providers, or no treatment(16)(8)(18)(9)(19). |
| Mass media exposure | 4 | Women exposed to mass media had about twice the odds of using allopathic treatment compared with those with no exposure. ​ Additional studies linked exposure to TV/radio/print with higher knowledge of RTI symptoms and greater probability of facility‑based care (9)(2)(19). |
| Distance to facility / transport | 5 | As distance to the nearest private facility increased, the probability of seeking treatment/consultation fell significantly; greater distance to government facilities also reduced utilisation. ​ RTI and ANC studies highlighted distance, lack of transport, and wage loss associated with travel as key barriers(8)(20)(15)(18). |
| Awareness / knowledge | 7 | Low knowledge about menarche and reproductive physiology was associated with poor care‑seeking for menstrual and gynaecological problems. In one RTI study, 83.1% of symptomatic women who did not seek care reported not knowing that treatment was needed. Many RTI studies documented symptom normalisation, poor recognition of risk, and lack of awareness about available services as dominant reasons for not seeking facility‑based care(12)(21)(2)(18)(19). |

**Supplementary Table 5: Determinants and factors associated with women not seeking treatment for Gynaecological morbidities.**

**
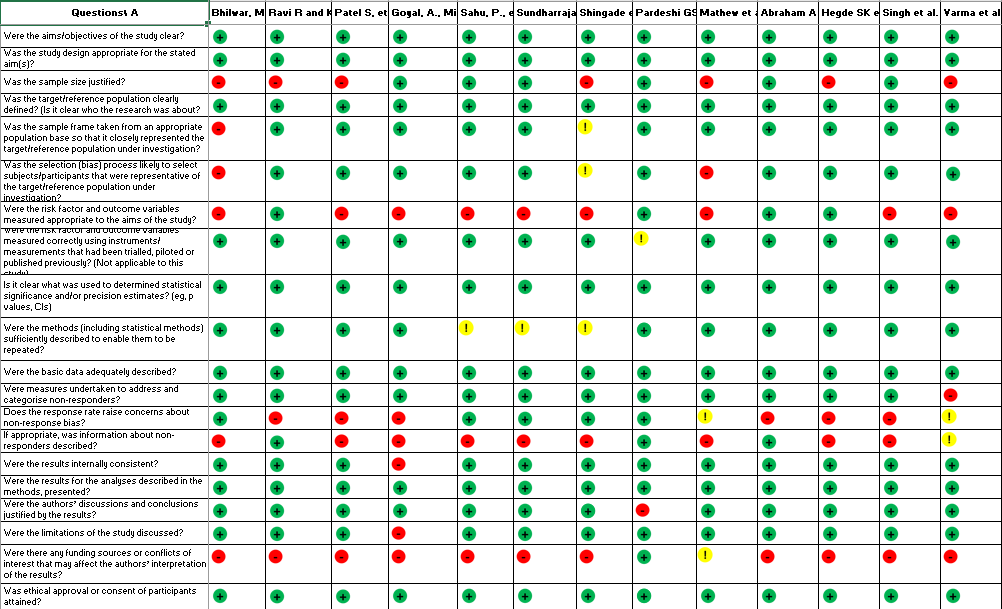
**

**Supplementary Figure 1. Graphical reporting of risk of bias in studies reporting on treatment seeking for reproductive morbidities**

48 studies are included for quantitative analysis and the below figure showing risk of bias in studies reporting on treatment seeking for reproductive morbidities are assessed using AXIS tool where ‘+’ symbol in green indicates YES and denotes Low risk of bias, ‘?’ in yellow indicate Do not Know and denotes ‘some concerns’ and ‘-‘symbol in red indicate NO and denotes ‘serious concerns’. The figure below provides the 20 questions for the studies.

**
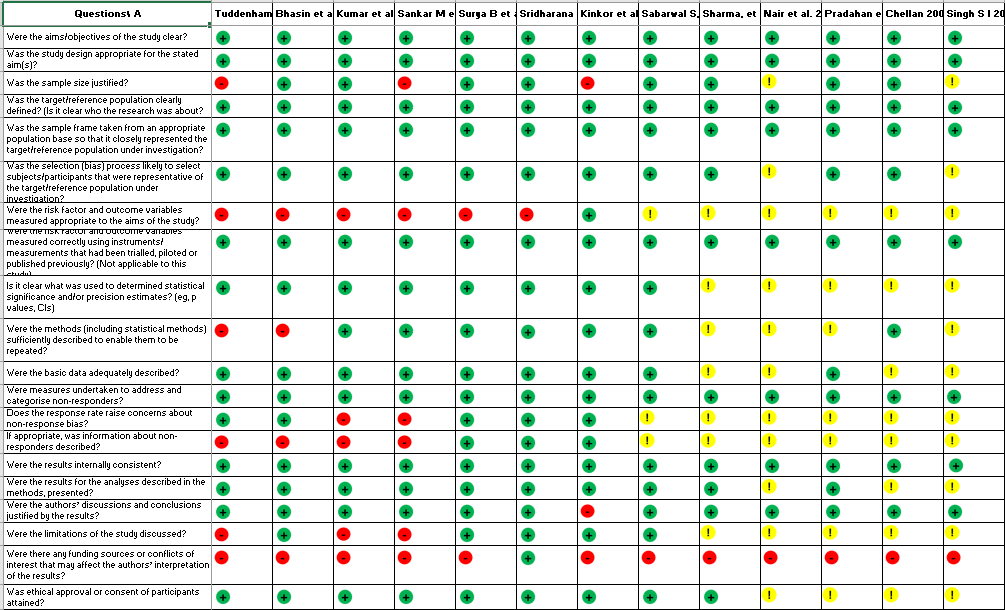
**

**Supplementary Figure 2. Graphical reporting of risk of bias in studies reporting on treatment seeking for reproductive morbidities**

48 studies are included for quantitative analysis and the below figure showing risk of bias in studies reporting on treatment seeking for reproductive morbidities are assessed using AXIS tool where ‘+’ symbol in green indicates YES and denotes Low risk of bias, ‘?’ in yellow indicate Do not Know and denotes ‘some concerns’ and ‘-‘symbol in red indicate NO and denotes ‘serious concerns’. The figure below provides the 20 questions for the studies.

**
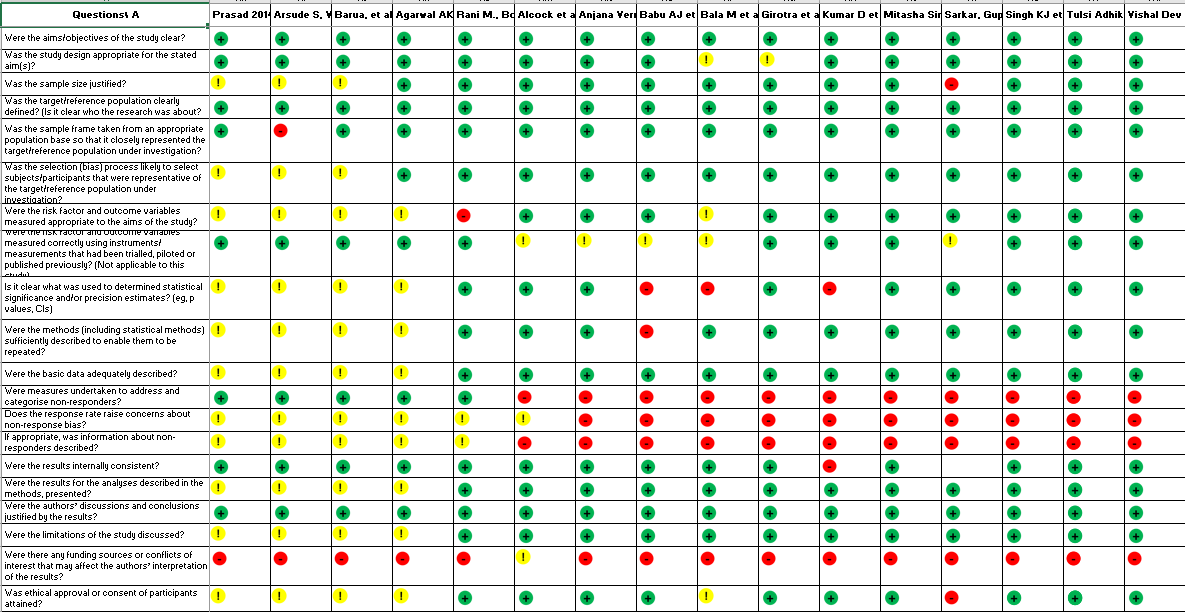
**

**Supplementary Figure 3. Graphical reporting of risk of bias in studies reporting on treatment seeking for reproductive morbidities**

48 studies are included for quantitative analysis and the below figure showing risk of bias in studies reporting on treatment seeking for reproductive morbidities are assessed using AXIS tool where ‘+’ symbol in green indicates YES and denotes Low risk of bias, ‘?’ in yellow indicate Do not Know and denotes ‘some concerns’ and ‘-‘symbol in red indicate NO and denotes ‘serious concerns’. The figure below provides the 20 questions for the studies.

**
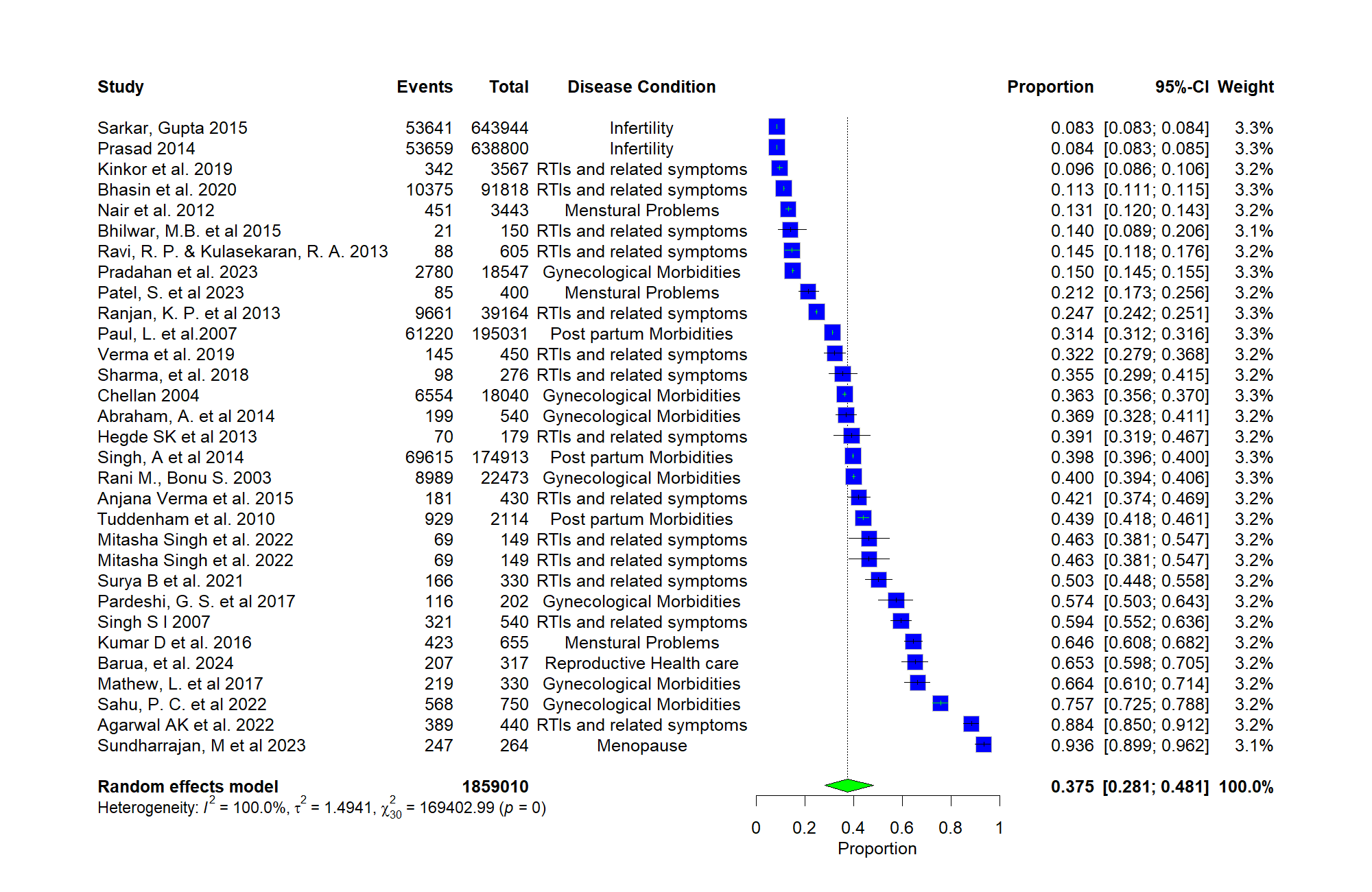
**

**Supplementary Figure 4: Forest plot showing pooled prevalence of gynaecological morbidities**


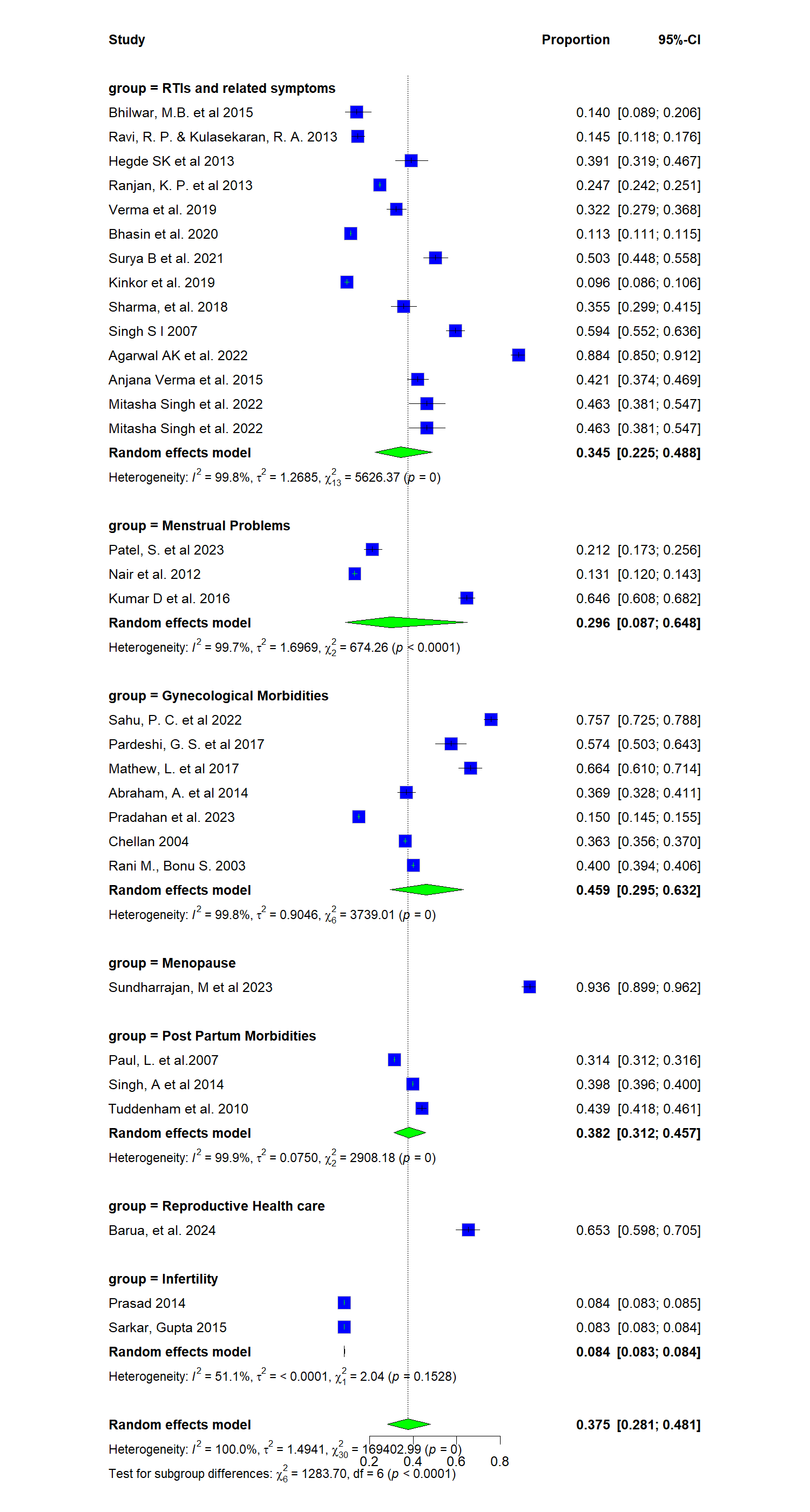


**Supplementary Figure 5: Forest plot showing pooled prevalence of gynaecological morbidities and sub-grouped based on their existing disease condition**


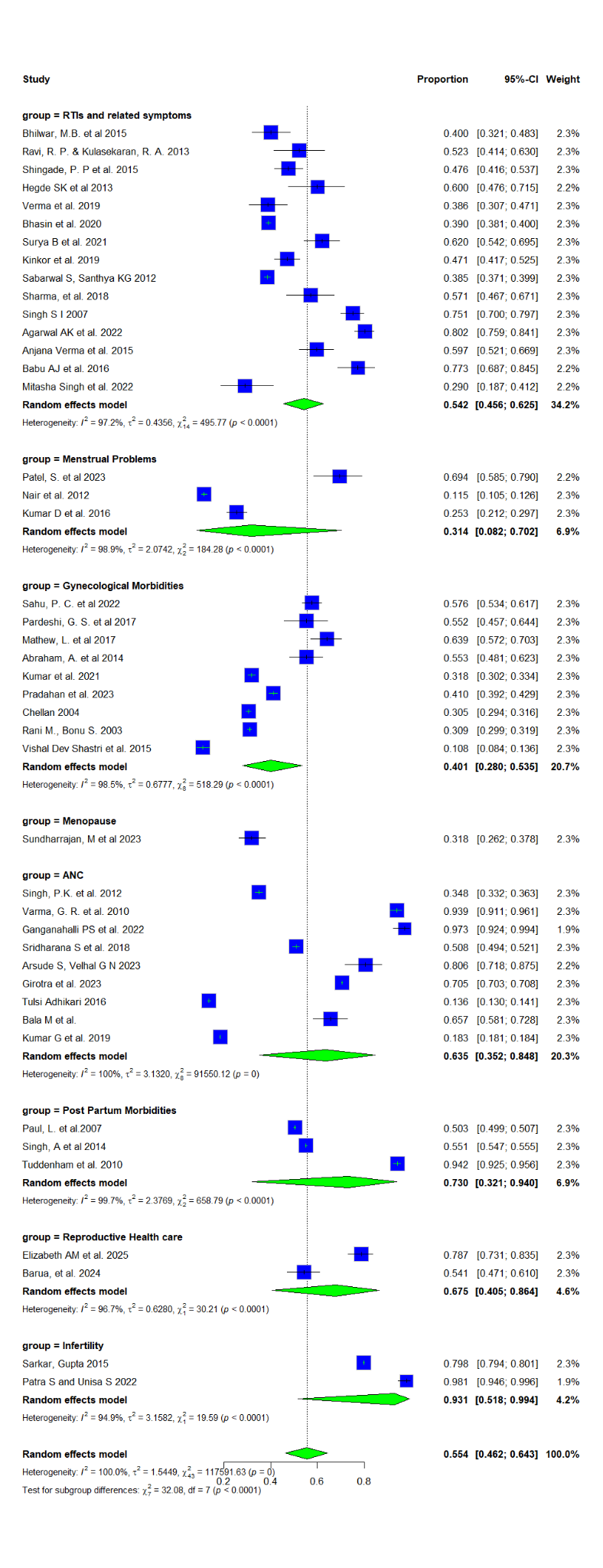


**Supplementary Figure 6. Forest plot showing pooled proportion of individuals who sought treatment and sub-grouped based on their existing disease condition**

The proportion of individual studies was determined by dividing the number of individuals who sought treatment by the total number of individuals exhibiting at least one symptom of the disease condition related to reproductive morbidity, and synthesized through meta-analysis employing the random effects Restricted Maximum Likelihood (REML) method. The studies were subsequently categorized according to the disease condition. Research on multimorbidity and self-reported illnesses encompasses individuals experiencing one or more reproductive morbidities, including reproductive tract infections (RTIs) and associated symptoms, menstrual disorders, gynaecological conditions, menopause, antenatal care (ANC), postpartum morbidities, reproductive healthcare, and infertility.


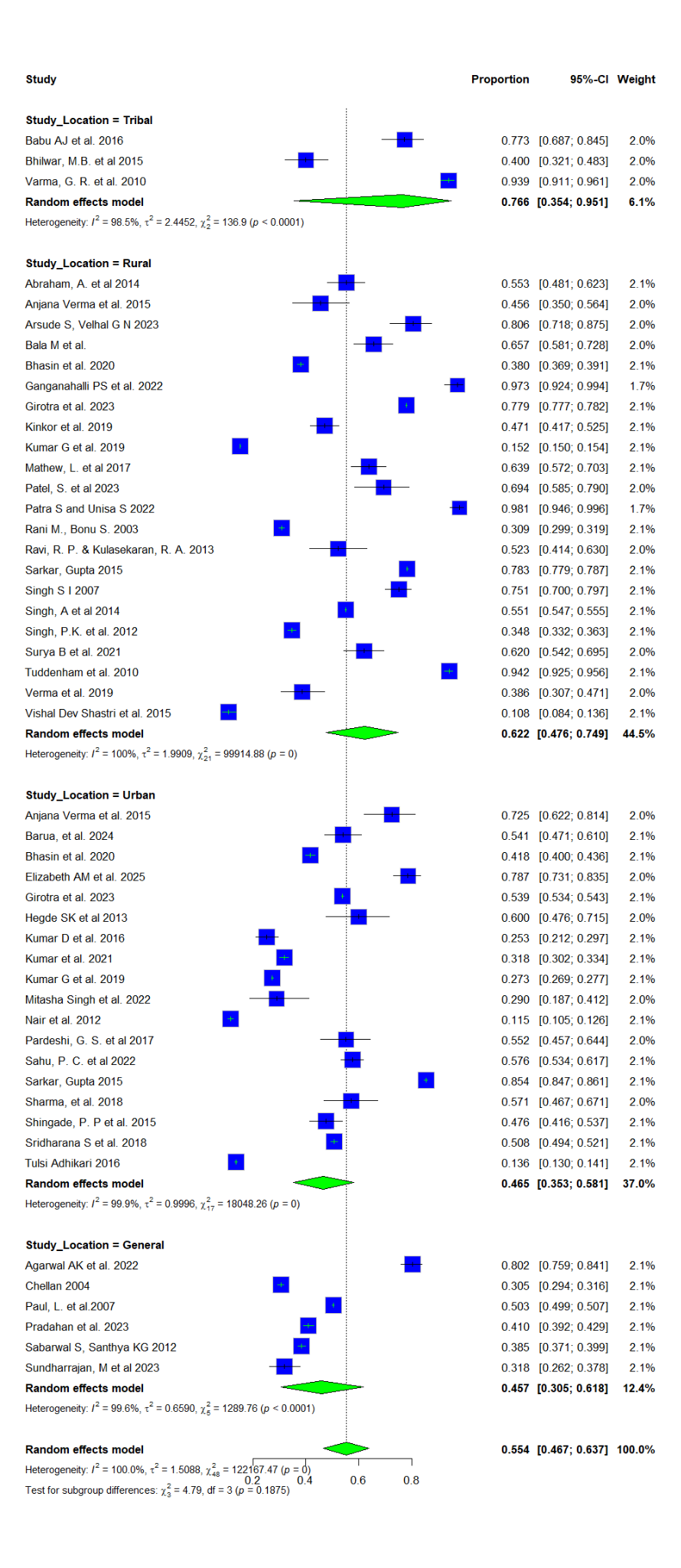


**Supplementary Figure 7: Forest plot of subgroup based on study setting of women who sought treatment**


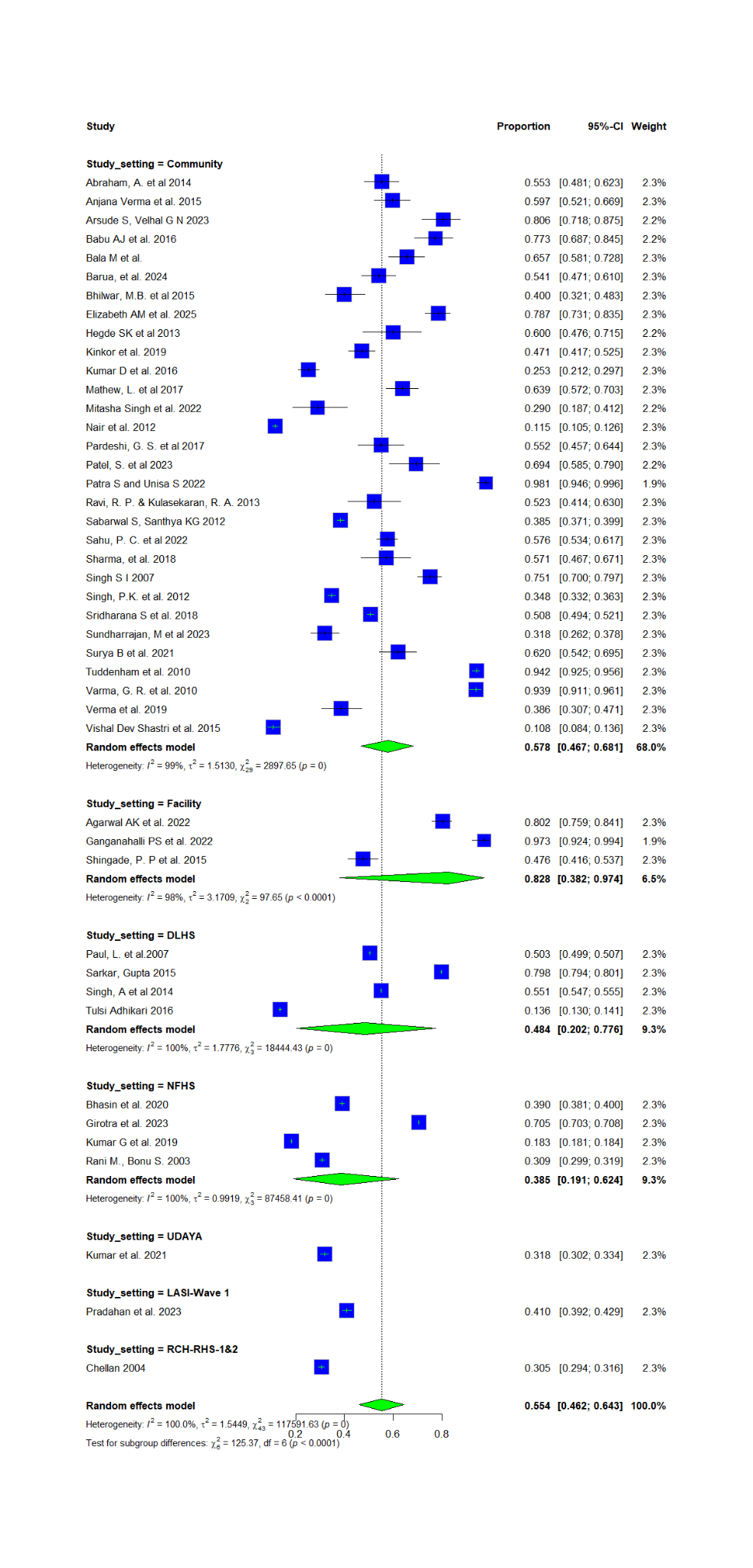


**Supplementary Figure 8: Forest plot of subgroup based on survey type of women who sought treatment**


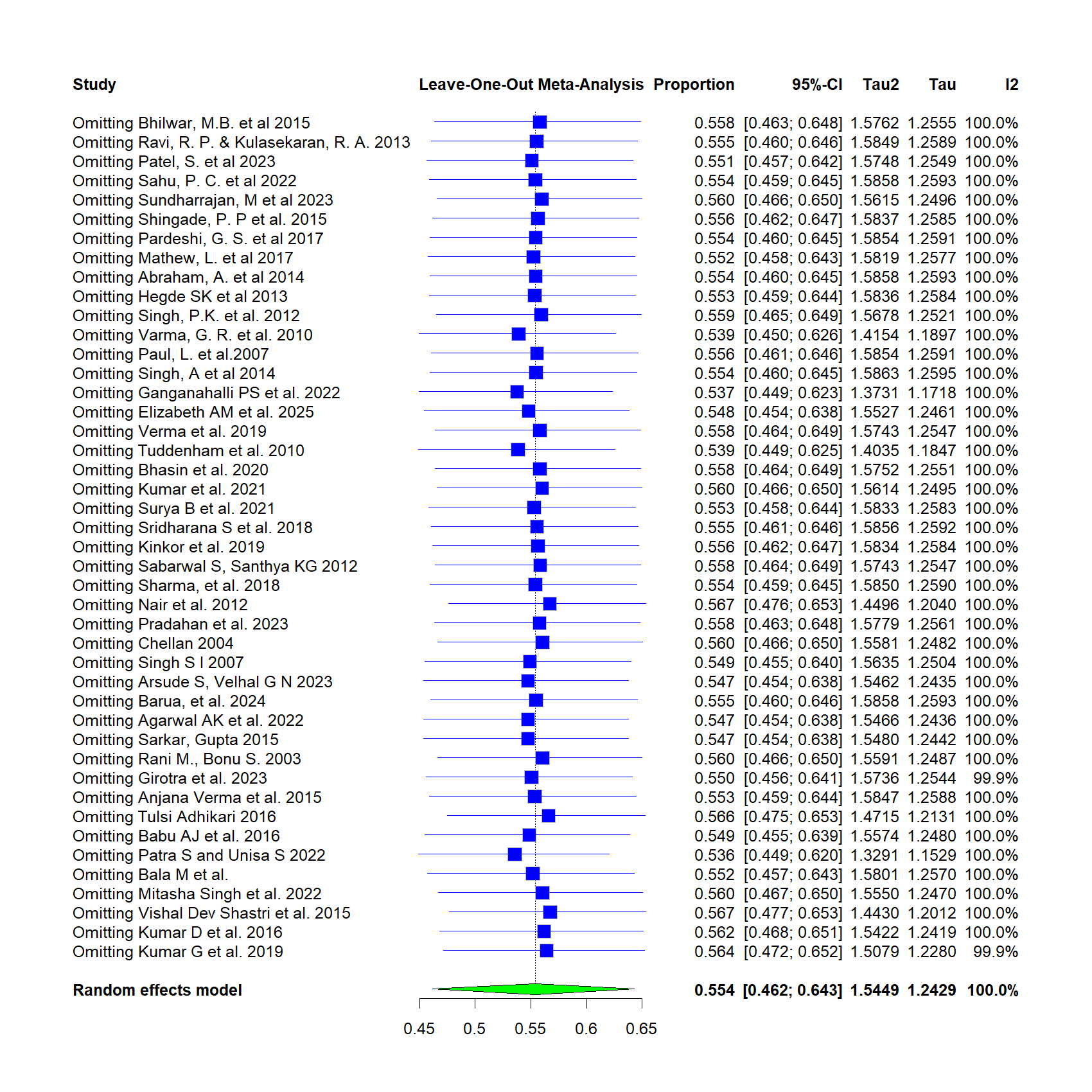


**Supplementary Figure 9. Leave-one-out sensitivity analysis for a pooled proportion of individuals who sought treatment for reproductive health-seeking behaviour among women.**


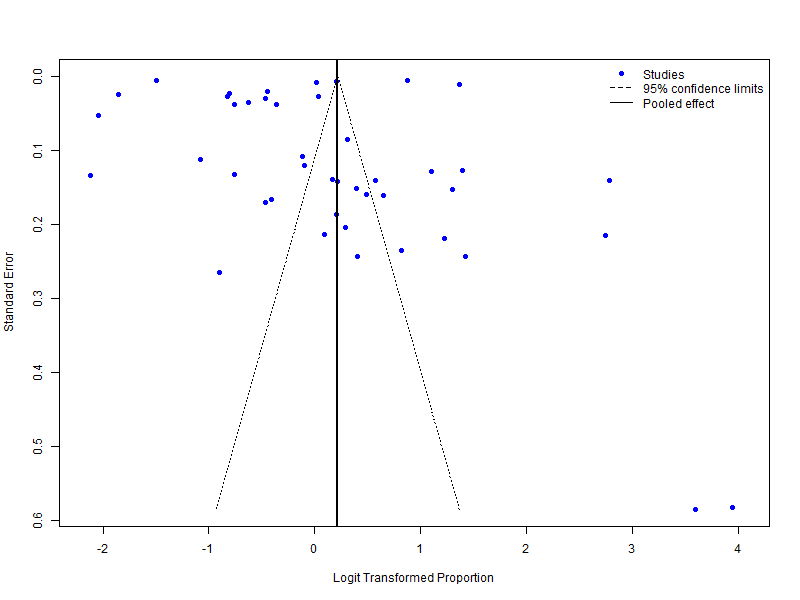


**Supplementary Figure 10: Funnel Plot showing Healthcare-Seeking for Reproductive Morbidities**


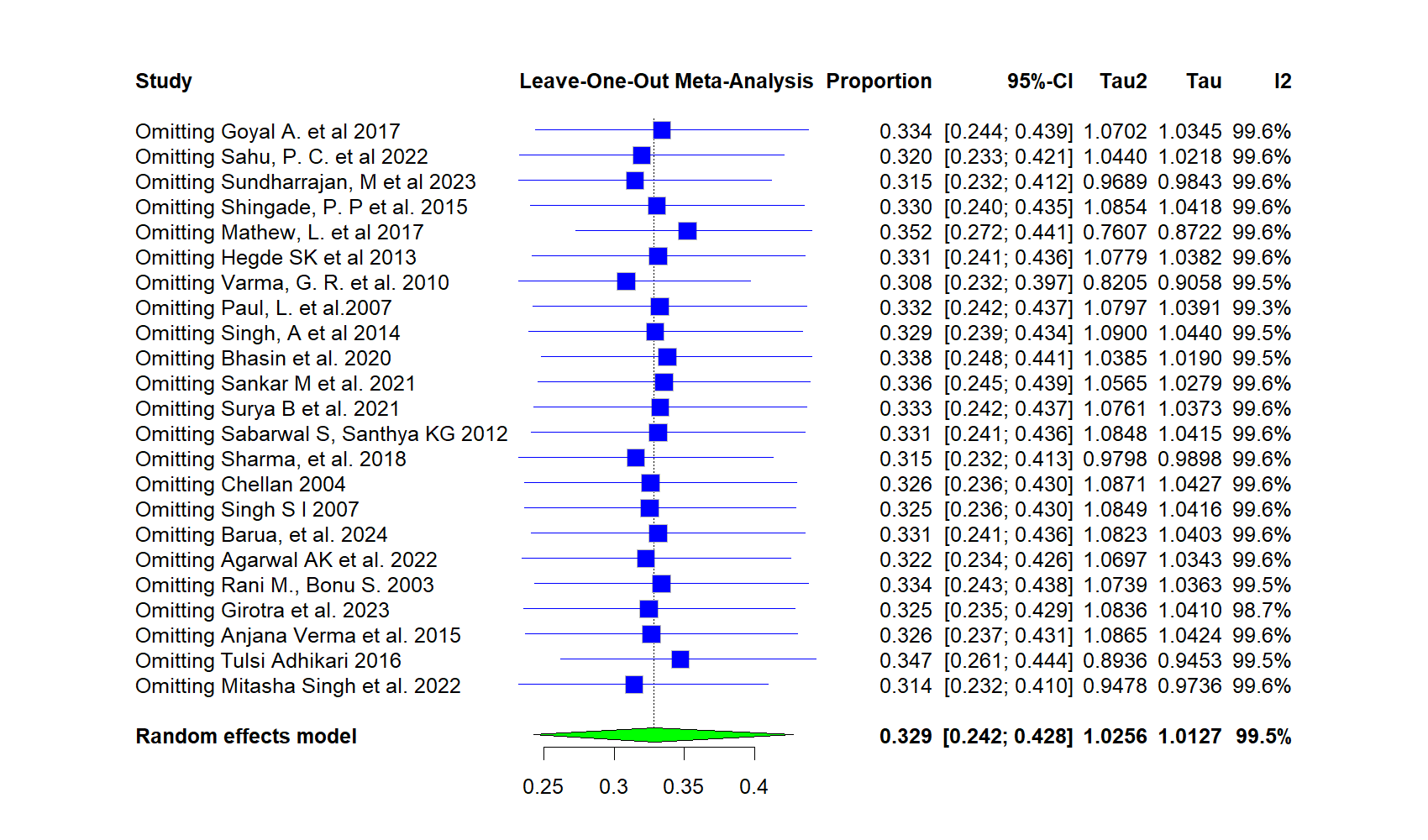


**Supplementary Figure 11. Leave-one-out analysis for individuals who sought treatment from government facility**

**
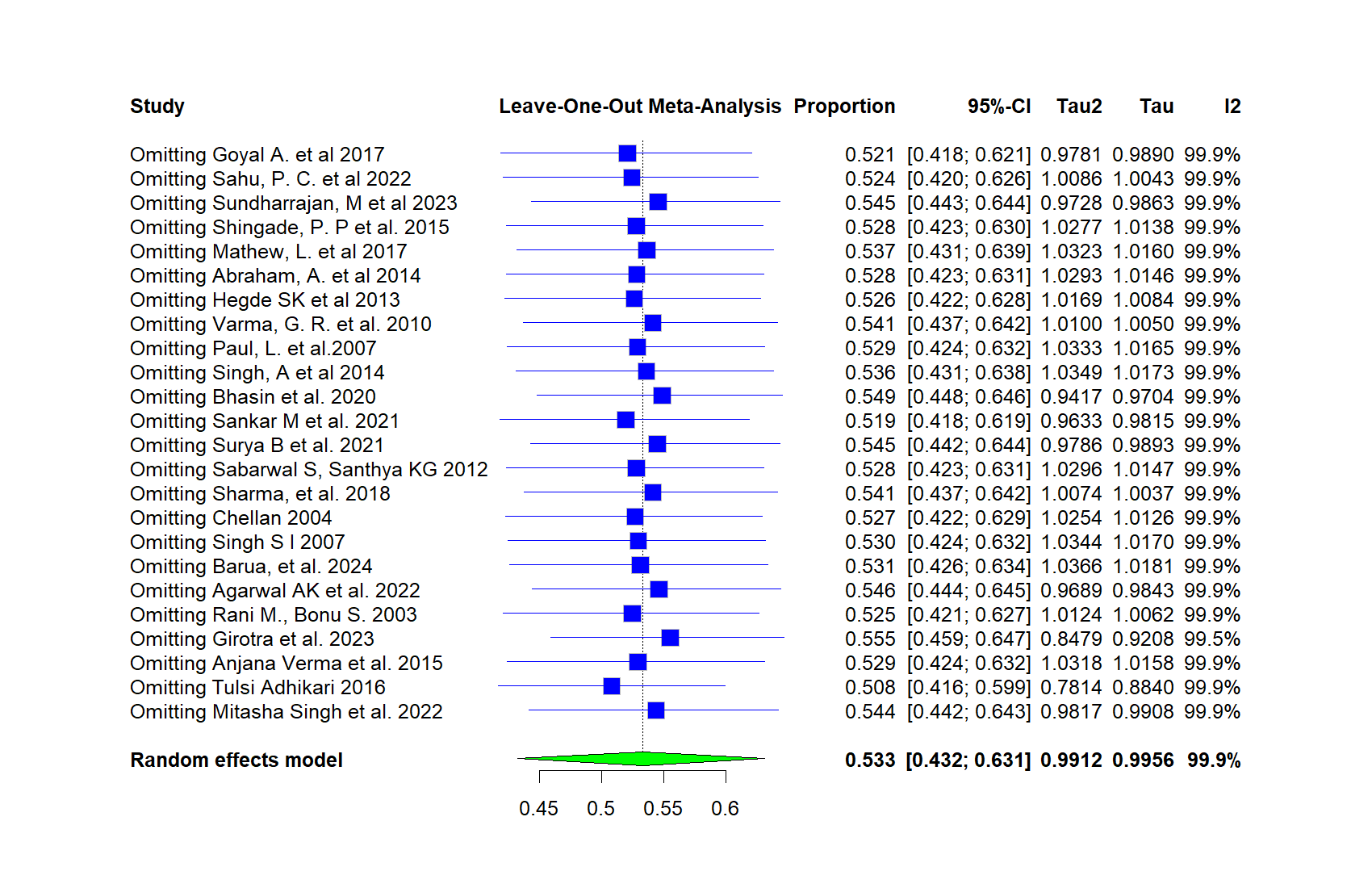
**

**Supplementary Figure 12. Leave-one-out sensitivity analysis for the pooled proportion of individuals who sought treatment from private facility.**


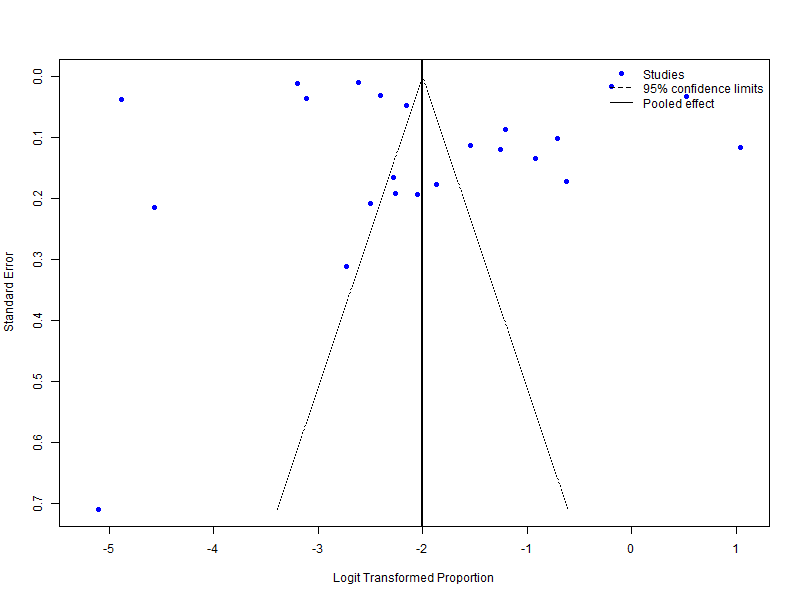


**Supplementary Figure 13: Funnel Plot showing Healthcare-Seeking for Reproductive Morbidities in Government Facilities**


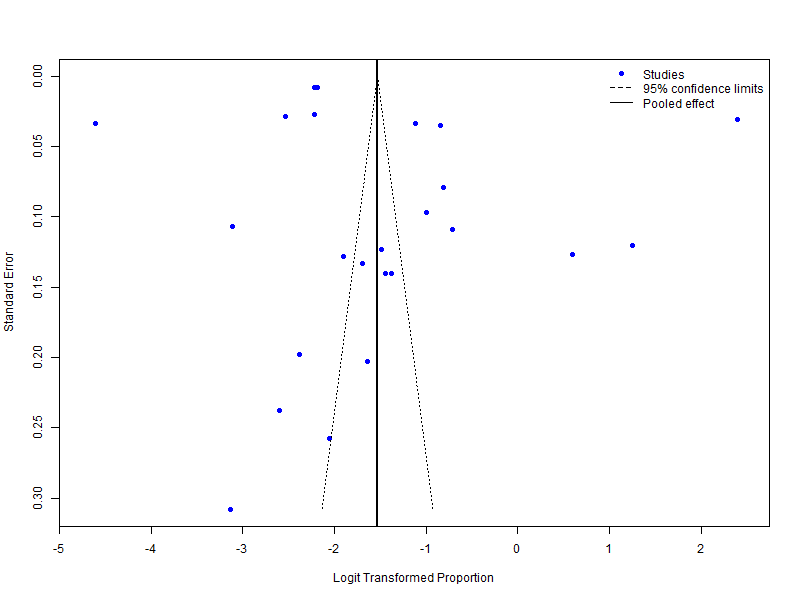


**Supplementary Figure 14: Funnel Plot of Healthcare-Seeking for Reproductive Morbidities in Private Facilities**


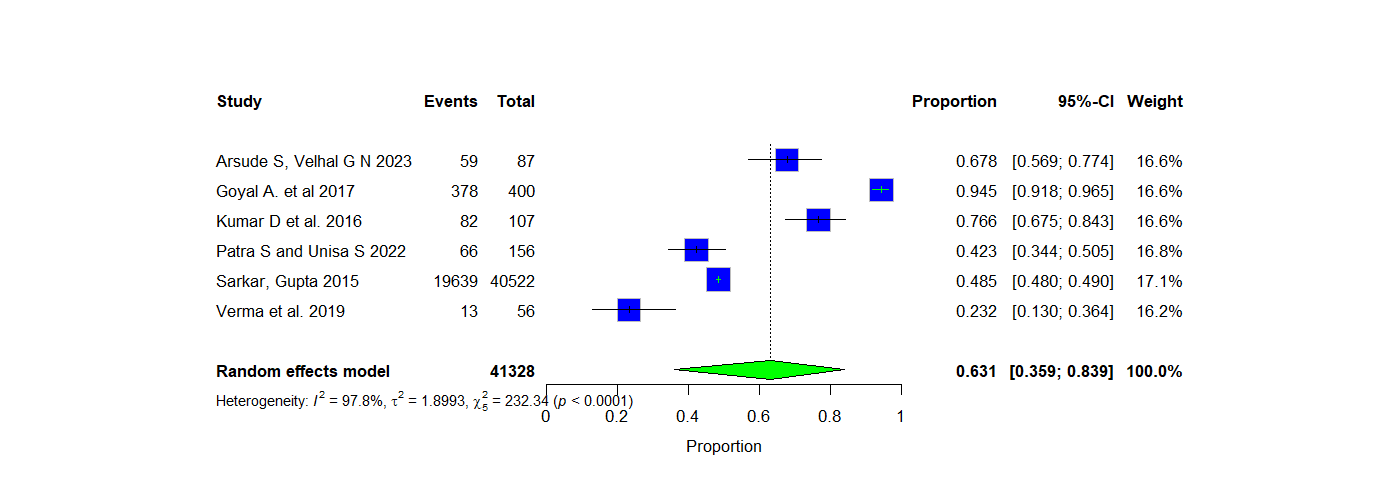


**Supplementary Figure 15: Forest plot of pooled proportion of women who sought Allopathy treatment**


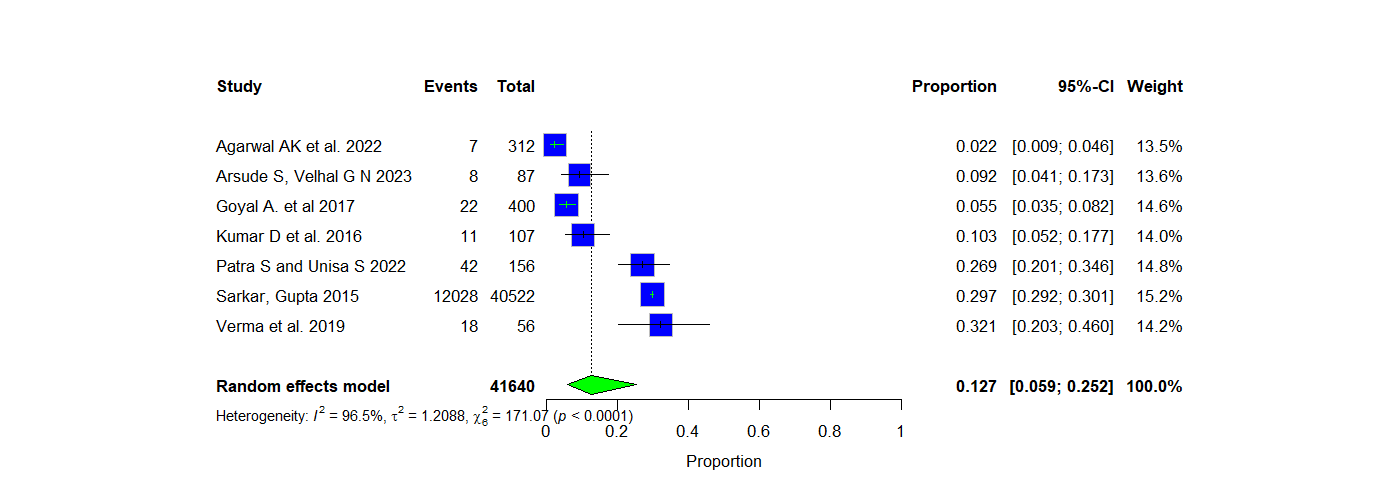


**Supplementary Figure 16: Forest plot of pooled proportion of women who sought Alternative medicine for treatment**


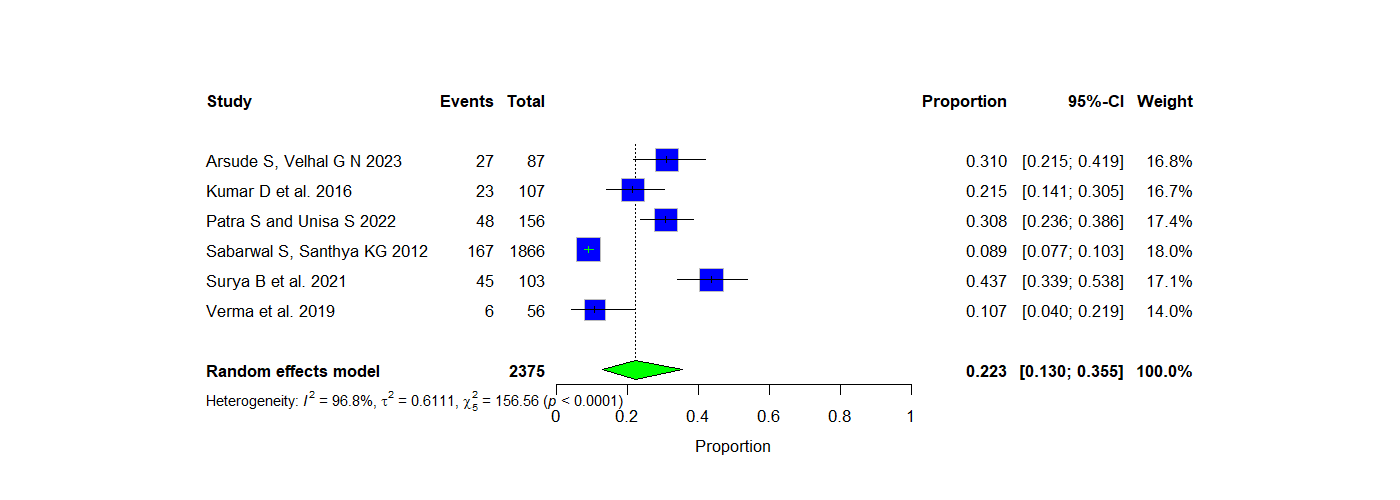


**Supplementary Figure 17: Forest plot of pooled proportion of women who sought treatment by home remedies**


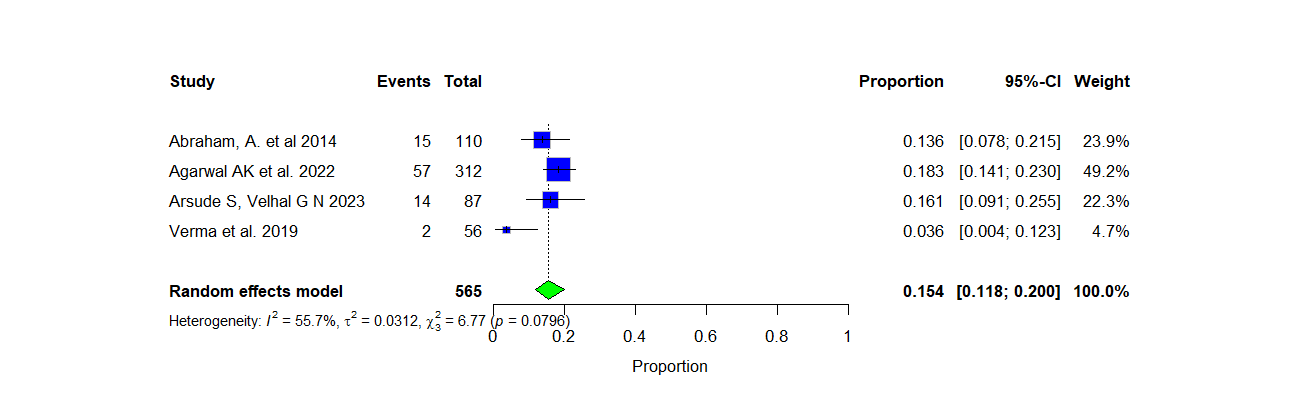


**Supplementary Figure 18: Forest plot of pooled proportion of women who sought treatment over the counter medicine**


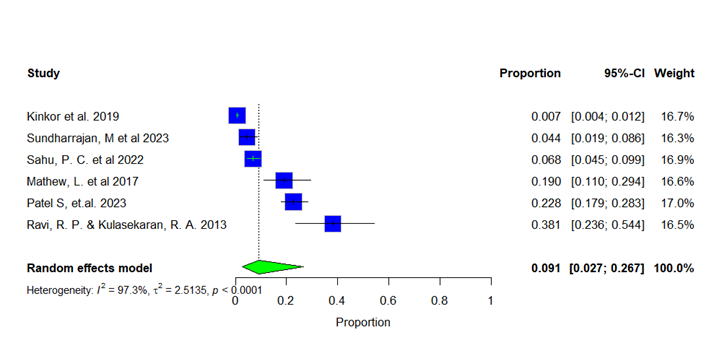


**Supplementary Figure 19. Forest plot showing pooled proportion of individuals who did not sought treatment due to high treatment expenses for existing disease condition**

Proportion from individual studies was calculated by dividing the number of individuals who considered ‘expensive treatment’ as a reason for not seeking healthcare with total number of individuals who did not seek treatment, and synthesized through meta-analysis employing the random effects Restricted Maximum Likelihood (REML) method.


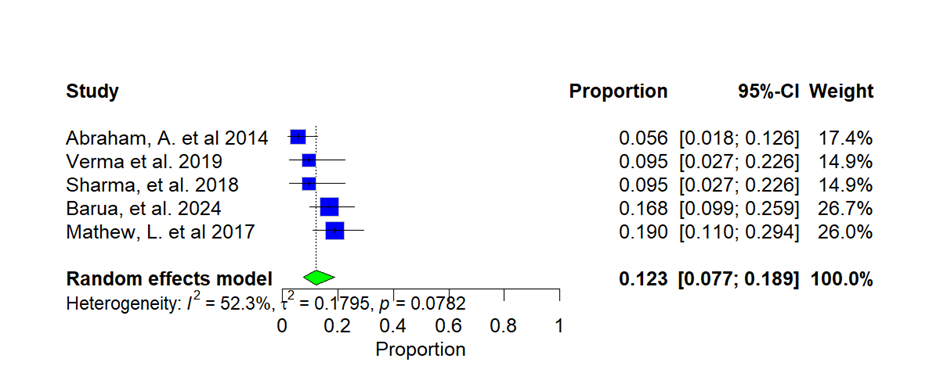


**Supplementary Figure 20. Forest plot showing pooled proportion of individuals who did not sought treatment due to financial constraint to get treated for existing disease condition**

Proportion from individual studies was calculated by dividing the number of individuals who considered ‘financial constraint’ as a reason for not seeking healthcare with total number of individuals who did not seek treatment, and synthesized through meta-analysis employing the random effects Restricted Maximum Likelihood (REML) method.


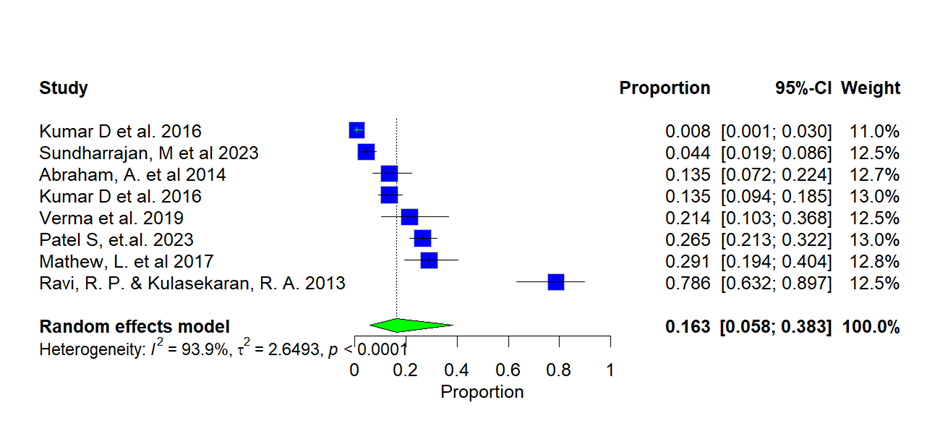


**Supplementary Figure 21. Forest plot showing pooled proportion of individuals who did not sought treatment due to shyness to discuss the existing disease condition**

Proportion from individual studies was calculated by dividing the number of individuals who considered ‘shyness’ as a reason for not seeking healthcare with total number of individuals who did not seek treatment, and synthesized through meta-analysis employing the random effects Restricted Maximum Likelihood (REML) method.


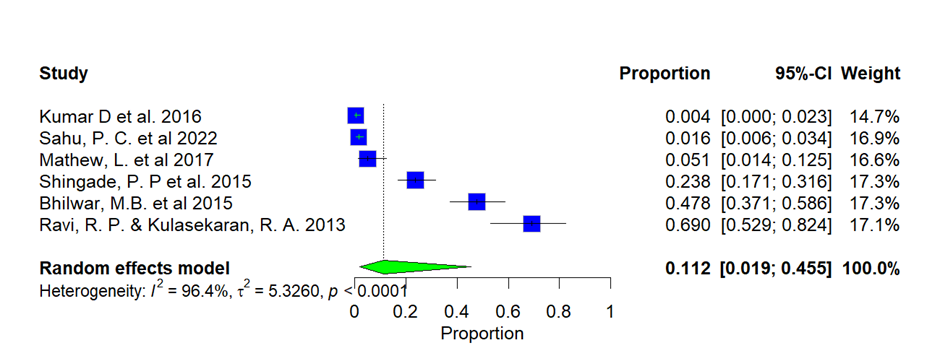


**Supplementary Figure 22. Forest plot showing pooled proportion of individuals who did not sought treatment due to unavailability of lady doctor in the health facility to discuss the existing disease condition**

Proportion from individual studies was calculated by dividing the number of individuals who considered ‘unavailability of lady doctor in the health facility’ as a reason for not seeking healthcare with total number of individuals who did not seek treatment, and synthesized through meta-analysis employing the random effects Restricted Maximum Likelihood (REML) method.


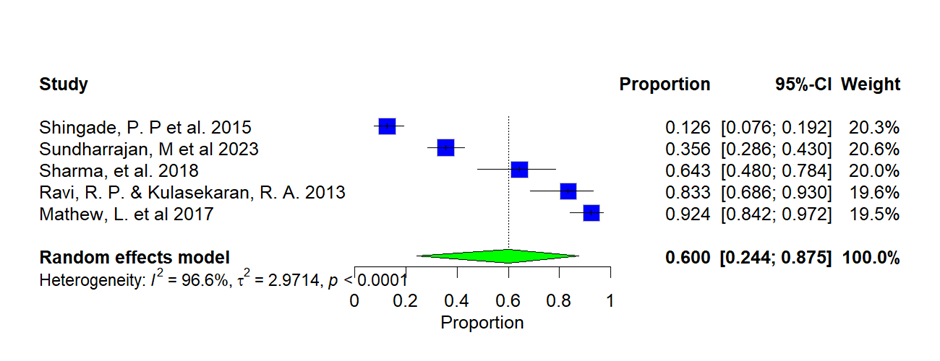


**Supplementary Figure 23. Forest plot showing pooled proportion of individuals who did not sought treatment, perceived as a normal condition**

Proportion from individual studies was calculated by dividing the number of individuals who considered ‘perceived as a normal condition’ as a reason for not seeking healthcare with total number of individuals who did not seek treatment, and synthesized through meta-analysis employing the random effects Restricted Maximum Likelihood (REML) method


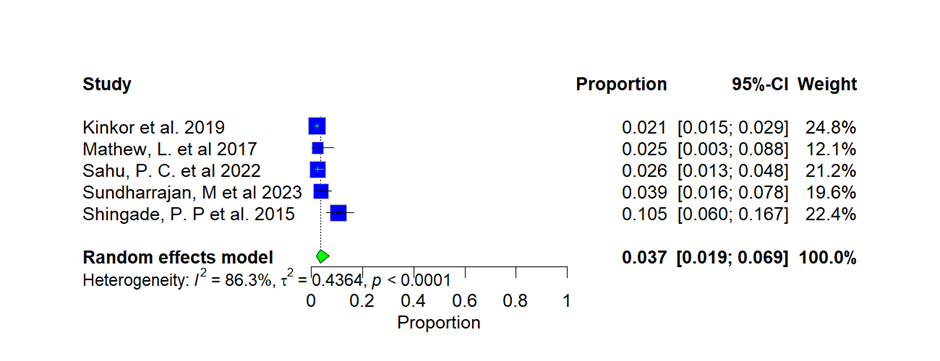


**Supplementary Figure 24. Forest plot showing pooled proportion of individuals who did not sought treatment due to family restrictions to go to the health facility getting treated for the existing disease condition**

Proportion from individual studies was calculated by dividing the number of individuals who considered ‘family restrictions to go to health facility’ as a reason for not seeking healthcare with total number of individuals who did not seek treatment, and synthesized through meta-analysis employing the random effects Restricted Maximum Likelihood (REML) method


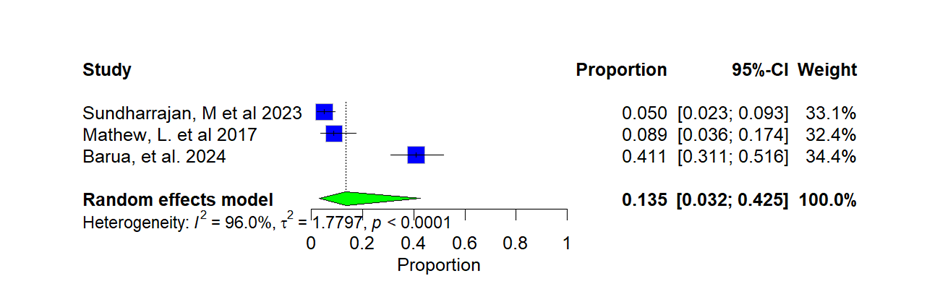


**Supplementary Figure 25. Forest plot showing pooled proportion of individuals who did not sought treatment due to lack of awareness about the existing disease condition**

Proportion from individual studies was calculated by dividing the number of individuals who considered ‘lack of awareness about the existing disease condition’ as a reason for not seeking healthcare with total number of individuals who did not seek treatment, and synthesized through meta-analysis employing the random effects Restricted Maximum Likelihood (REML) method


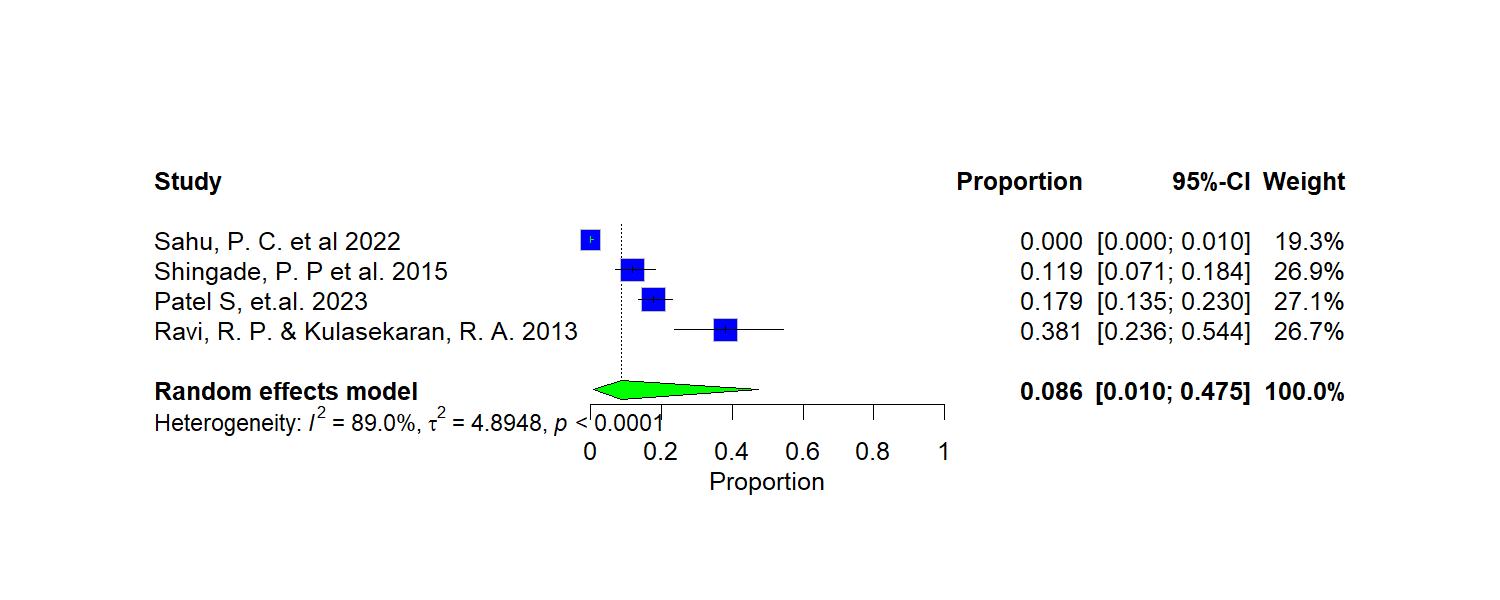


**Supplementary Figure 26. Forest plot showing pooled proportion of individuals who did not sought treatment due to poor healthcare services for the existing disease condition**

Proportion from individual studies was calculated by dividing the number of individuals who considered ‘poor healthcare services’ as a reason for not seeking healthcare with total number of individuals who did not seek treatment, and synthesized through meta-analysis employing the random effects Restricted Maximum Likelihood (REML) method


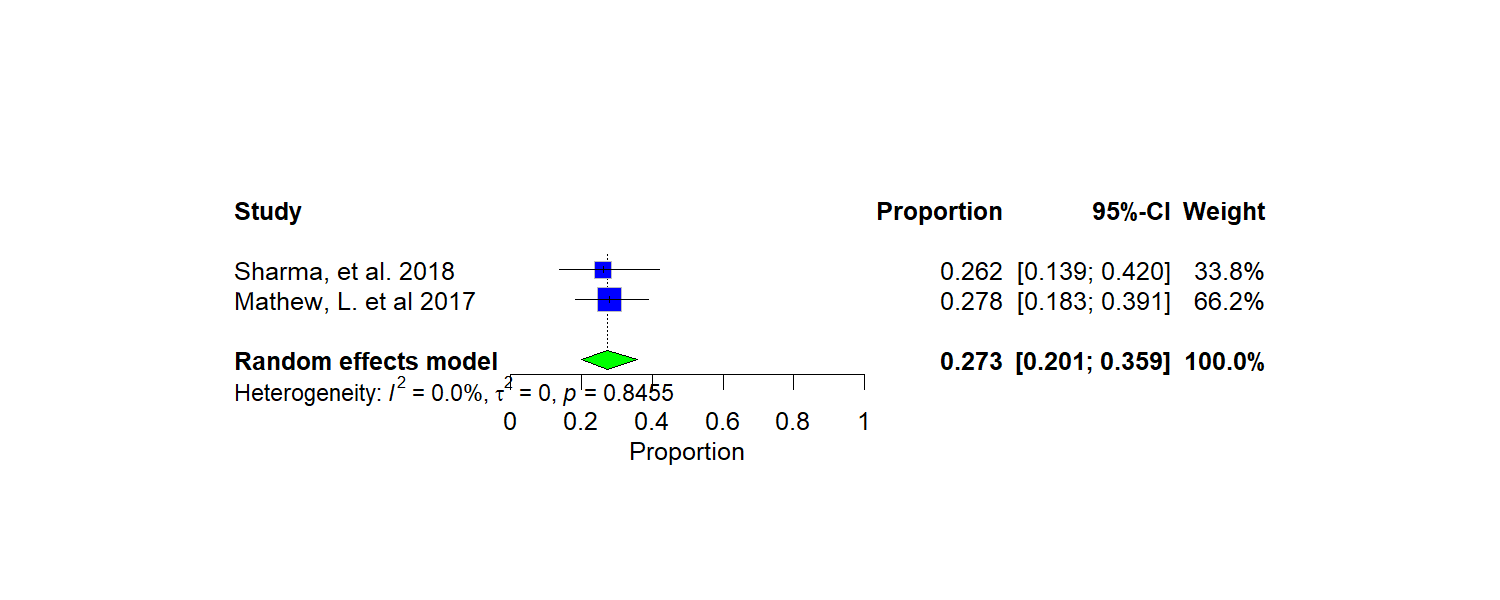


**Supplementary Figure 27. Forest plot showing pooled proportion of individuals who did not sought treatment due to communication barrier to discuss with the doctor for the existing disease condition**

Proportion from individual studies was calculated by dividing the number of individuals who considered ‘communication barrier to discuss with the doctor’ as a reason for not seeking healthcare with total number of individuals who did not seek treatment, and synthesized through meta-analysis employing the random effects Restricted Maximum Likelihood (REML) method


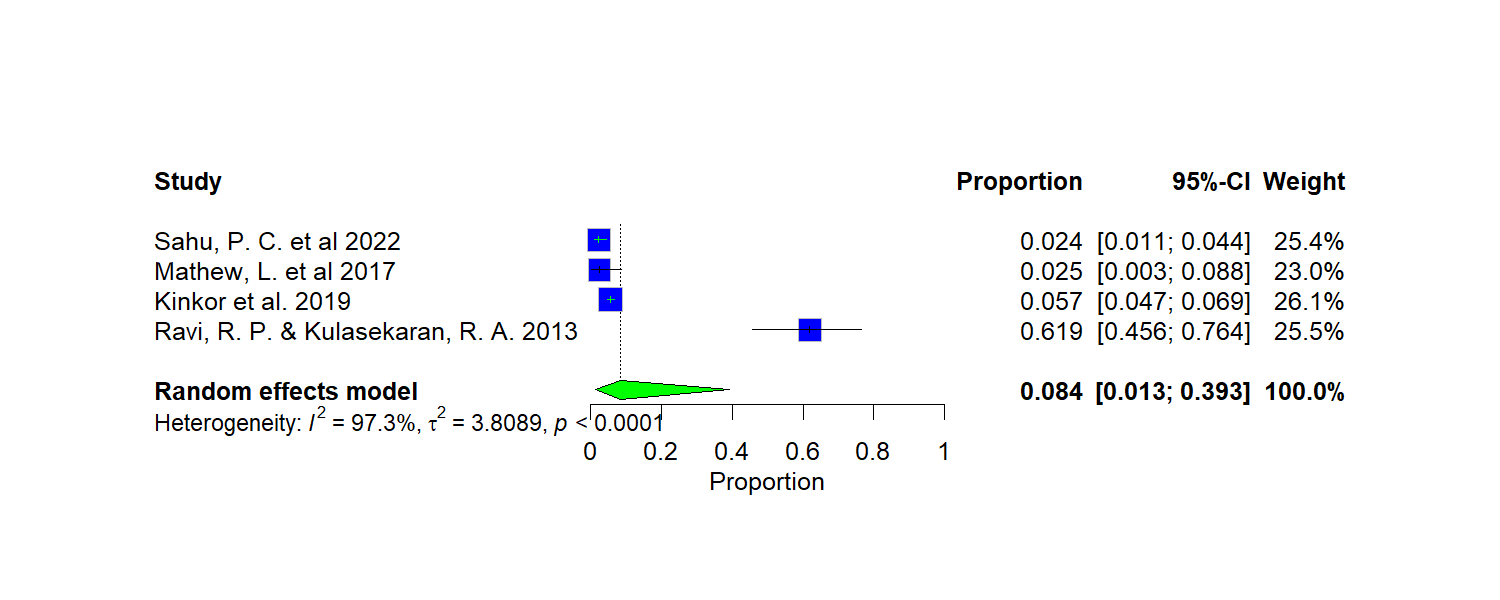


**Supplementary Figure 28. Forest plot showing pooled proportion of individuals who did not sought treatment due to the distance from residence to the health facility is far to get treated for the existing disease condition**

Proportion from individual studies was calculated by dividing the number of individuals who considered ‘the distance from residence to the health facility is far to get treated’ as a reason for not seeking healthcare with total number of individuals who did not seek treatment, and synthesized through meta-analysis employing the random effects Restricted Maximum Likelihood (REML) method


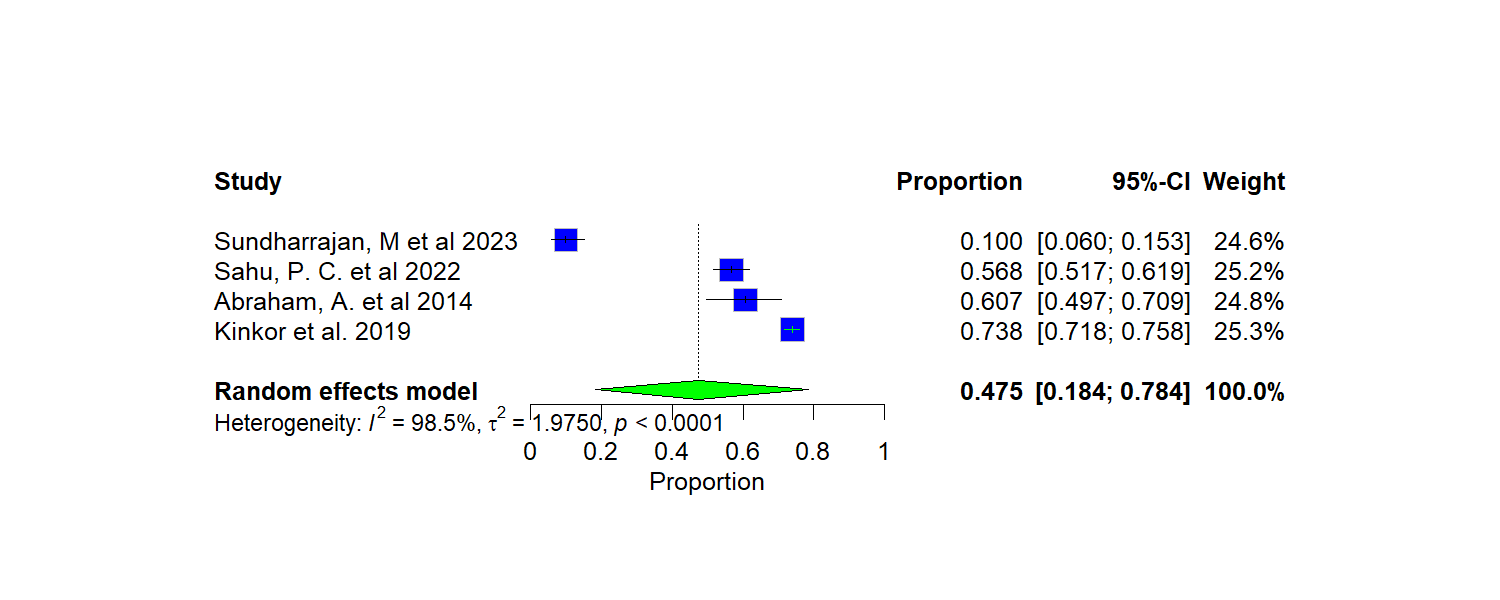


**Supplementary Figure 29. Forest plot showing pooled proportion of individuals who did not sought treatment due to perception of no need for treatment for the existing disease condition**

Proportion from individual studies was calculated by dividing the number of individuals who considered ‘perception of no need for treatment’ as a reason for not seeking healthcare with total number of individuals who did not seek treatment, and synthesized through meta-analysis employing the random effects Restricted Maximum Likelihood (REML) method


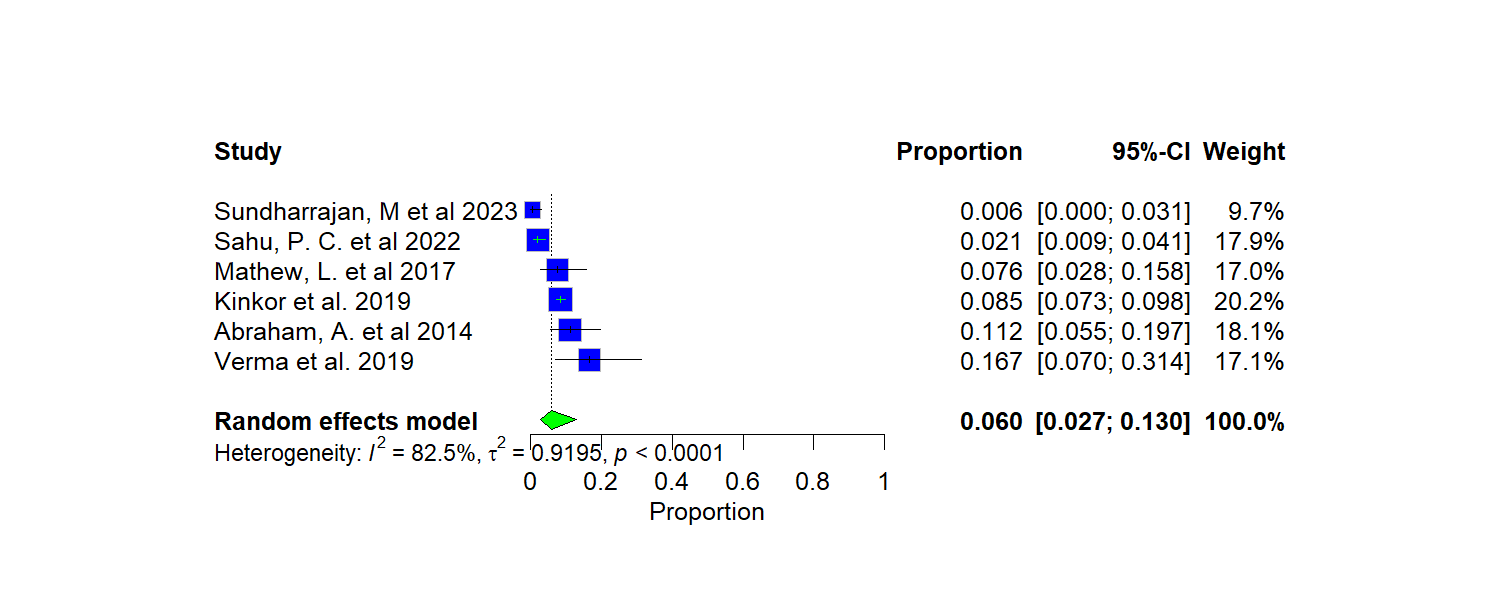


**Supplementary Figure 30. Forest plot showing pooled proportion of individuals who did not sought treatment due to lack of time to get treated for the existing disease condition**

Proportion from individual studies was calculated by dividing the number of individuals who considered ‘lack of time to get treated’ as a reason for not seeking healthcare with total number of individuals who did not seek treatment, and synthesized through meta-analysis employing the random effects Restricted Maximum Likelihood (REML) method


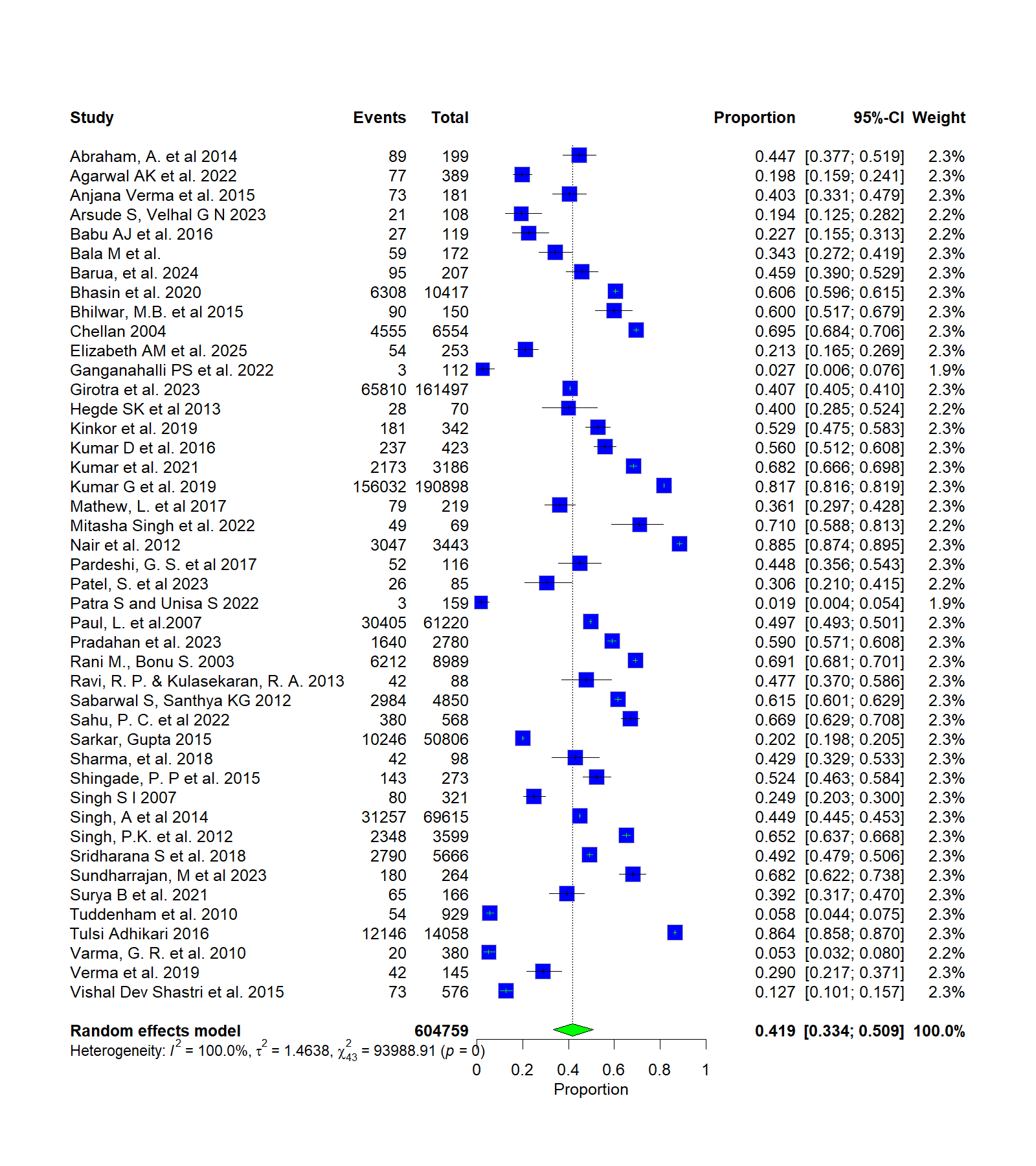


**Supplementary Figure F31: Forest plot of pooled proportion of women who did not sought treatment**

PRISMA 2020 Checklist

| **Section and Topic** | **Item #** | **Checklist item** | **Location where item is reported** |
| --- | --- | --- | --- |
| **TITLE** | | |  |
| Title | 1 | Identify the report as a systematic review. | Manuscript Page 1 |
| **ABSTRACT** | | |  |
| Abstract | 2 | See the PRISMA 2020 for Abstracts checklist. | Manuscript Page 2-3 |
| **INTRODUCTION** | | |  |
| Rationale | 3 | Describe the rationale for the review in the context of existing knowledge. | Manuscript Page 4 |
| Objectives | 4 | Provide an explicit statement of the objective(s) or question(s) the review addresses. | Manuscript Page 4 |
| **METHODS** | | |  |
| Eligibility criteria | 5 | Specify the inclusion and exclusion criteria for the review and how studies were grouped for the syntheses. | Manuscript Page 5 |
| Information sources | 6 | Specify all databases, registers, websites, organisations, reference lists and other sources searched or consulted to identify studies. Specify the date when each source was last searched or consulted. | Manuscript Page 5 |
| Search strategy | 7 | Present the full search strategies for all databases, registers and websites, including any filters and limits used. | Supplementary Material Page 2-4 |
| Selection process | 8 | Specify the methods used to decide whether a study met the inclusion criteria of the review, including how many reviewers screened each record and each report retrieved, whether they worked independently, and if applicable, details of automation tools used in the process. | Manuscript Page 6 |
| Data collection process | 9 | Specify the methods used to collect data from reports, including how many reviewers collected data from each report, whether they worked independently, any processes for obtaining or confirming data from study investigators, and if applicable, details of automation tools used in the process. | Manuscript Page 6 |
| Data items | 10a | List and define all outcomes for which data were sought. Specify whether all results that were compatible with each outcome domain in each study were sought (e.g. for all measures, time points, analyses), and if not, the methods used to decide which results to collect. | Manuscript Page 6 |
|  | 10b | List and define all other variables for which data were sought (e.g. participant and intervention characteristics, funding sources). Describe any assumptions made about any missing or unclear information. | Manuscript Page 6 |
| Study risk of bias assessment | 11 | Specify the methods used to assess risk of bias in the included studies, including details of the tool(s) used, how many reviewers assessed each study and whether they worked independently, and if applicable, details of automation tools used in the process. | Manuscript Page 6&7 |
| Effect measures | 12 | Specify for each outcome the effect measure(s) (e.g. risk ratio, mean difference) used in the synthesis or presentation of results. | Manuscript Page 6&7 |
| Synthesis methods | 13a | Describe the processes used to decide which studies were eligible for each synthesis (e.g. tabulating the study intervention characteristics and comparing against the planned groups for each synthesis (item #5)). | Manuscript Page 6 |
|  | 13b | Describe any methods required to prepare the data for presentation or synthesis, such as handling of missing summary statistics, or data conversions. | Manuscript Page 6 |
|  | 13c | Describe any methods used to tabulate or visually display results of individual studies and syntheses. | Manuscript Page 7 |
|  | 13d | Describe any methods used to synthesize results and provide a rationale for the choice(s). If meta-analysis was performed, describe the model(s), method(s) to identify the presence and extent of statistical heterogeneity, and software package(s) used. | Manuscript Page 7 |
|  | 13e | Describe any methods used to explore possible causes of heterogeneity among study results (e.g. subgroup analysis, meta-regression). | Manuscript Page 7 |
|  | 13f | Describe any sensitivity analyses conducted to assess robustness of the synthesized results. | Manuscript Page 7 |
| Reporting bias assessment | 14 | Describe any methods used to assess risk of bias due to missing results in a synthesis (arising from reporting biases). | Manuscript Page 8 |
| Certainty assessment | 15 | Describe any methods used to assess certainty (or confidence) in the body of evidence for an outcome. | NA |
| **RESULTS** | | |  |
| Study selection | 16a | Describe the results of the search and selection process, from the number of records identified in the search to the number of studies included in the review, ideally using a flow diagram. | Manuscript Page 8 |
|  | 16b | Cite studies that might appear to meet the inclusion criteria, but which were excluded, and explain why they were excluded. | Manuscript Page 8 |
| Study characteristics | 17 | Cite each included study and present its characteristics. | Manuscript table1 |
| Risk of bias in studies | 18 | Present assessments of risk of bias for each included study. | Manuscript Page 8 |
| Results of individual studies | 19 | For all outcomes, present, for each study: (a) summary statistics for each group (where appropriate) and (b) an effect estimate and its precision (e.g. confidence/credible interval), ideally using structured tables or plots. | Manuscript Page 9 |
| Results of syntheses | 20a | For each synthesis, briefly summarise the characteristics and risk of bias among contributing studies. | Manuscript Page 9 |
|  | 20b | Present results of all statistical syntheses conducted. If meta-analysis was done, present for each the summary estimate and its precision (e.g. confidence/credible interval) and measures of statistical heterogeneity. If comparing groups, describe the direction of the effect. | Manuscript Page 9-12 |
|  | 20c | Present results of all investigations of possible causes of heterogeneity among study results. | Manuscript Page 9-12 |
|  | 20d | Present results of all sensitivity analyses conducted to assess the robustness of the synthesized results. | Manuscript Page 9-12 |
| Reporting biases | 21 | Present assessments of risk of bias due to missing results (arising from reporting biases) for each synthesis assessed. | Manuscript Page 8 |
| Certainty of evidence | 22 | Present assessments of certainty (or confidence) in the body of evidence for each outcome assessed. | NA |
| **DISCUSSION** | | |  |
| Discussion | 23a | Provide a general interpretation of the results in the context of other evidence. | Manuscript Page 12-13 |
|  | 23b | Discuss any limitations of the evidence included in the review. | Manuscript Page 13-15 |
|  | 23c | Discuss any limitations of the review processes used. | Manuscript Page 13-15 |
|  | 23d | Discuss implications of the results for practice, policy, and future research. |  |
| **OTHER INFORMATION** | | |  |
| Registration and protocol | 24a | Provide registration information for the review, including register name and registration number, or state that the review was not registered. | Manuscript Page 2 |
|  | 24b | Indicate where the review protocol can be accessed, or state that a protocol was not prepared. | Manuscript Page 5 |
|  | 24c | Describe and explain any amendments to information provided at registration or in the protocol. | NA |
| Support | 25 | Describe sources of financial or non-financial support for the review, and the role of the funders or sponsors in the review. | Manuscript Page 16 |
| Competing interests | 26 | Declare any competing interests of review authors. | Manuscript Page 16 |
| Availability of data, code and other materials | 27 | Report which of the following are publicly available and where they can be found: template data collection forms; data extracted from included studies; data used for all analyses; analytic code; any other materials used in the review. | Manuscript table1 |

*From:*  Page MJ, McKenzie JE, Bossuyt PM, Boutron I, Hoffmann TC, Mulrow CD, et al. The PRISMA 2020 statement: an updated guideline for reporting systematic reviews. BMJ 2021;372:n71. doi: 10.1136/bmj.n71

**References:**

1. Agarwal AK, Mishra J, Verma PK, Mahore R, Verma R. Knowledge, attitude and treatment seeking behaviour for reproductive tract infections (RTI) and sexually transmitted infections (STIs) among married women …. gjmedph.com; 2022.

2. Bhasin S, Shukla A, Desai S. Services for women’s sexual and reproductive health in India: an analysis of treatment-seeking for symptoms of reproductive tract infections in a nationally representative survey. BMC Womens Health [Internet]. 2020;20(1):156. Available from: https://pubmed.ncbi.nlm.nih.gov/32723377/

3. Hegde S, Agrawal T, Ramesh N, Sugara M, Joseph P, Singh S, et al. Reproductive tract infections among women in a peri-urban under privileged area in Bangalore, India: Knowledge, prevalence, and treatment seeking behavior. Ann Trop Med Public Heal [Internet]. 2013;6(2):215–20. Available from: https://www.scopus.com/inward/record.uri?eid=2-s2.0-84883615734&doi=10.4103%2F1755-6783.116514&partnerID=40&md5=47477084a60bf74d4538401e57f58a5e

4. Sabarwal S, KG S. Treatment-seeking for symptoms of reproductive tract infections among young women in India. Int Perspect Sex Reprod Health [Internet]. 2012;38(2):90–8. Available from: https://pubmed.ncbi.nlm.nih.gov/22832149/

5. Bala M, Mane AB, Dohare S, Mahajan H, Rao E V, Dohare SK. A study of antenatal care practices among mothers in rural Gautam Budh Nagar, Uttar Pradesh. Indian J Public Heal Res Dev [Internet]. 2013;4(4):70–4. Available from: https://www.scopus.com/inward/record.uri?eid=2-s2.0-84887429098&doi=10.5958%2Fj.0976-5506.4.4.144&partnerID=40&md5=9654bc2a6db079c0640b17718edc4da8

6. Alcock G, Das S, N SM, Hate K, More S, Pantvaidya S, et al. Examining inequalities in uptake of maternal health care and choice of provider in underserved urban areas of Mumbai, India: a mixed methods study. BMC Pregnancy Childbirth [Internet]. 2015;15:231. Available from: https://pubmed.ncbi.nlm.nih.gov/26416081/

7. Rani M, Bonu S. Rural Indian women’s care-seeking behavior and choice of provider for gynecological symptoms. [Internet]. Vol. 34, Studies in family planning. United States; 2003. p. 173–85. Available from: https://pubmed.ncbi.nlm.nih.gov/14558320/

8. Babu A, Radha S, Nambisan B, Brahmanandan M. Gynaecological morbidities and health seeking behaviour of aged tribal women in Trivandrum district, Kerala, India. Int J Community Med Public Heal. 2016.

9. Ravi RP, Kulasekaran RA. Trends in Reproductive Tract Infections and Barriers to Seeking Treatment among Young Women: A Community Based Cross Sectional Study in South India. Am J Epidemiol Infect Dis [Internet]. 2013 Dec 7;1(4):53–8. Available from: http://pubs.sciepub.com/ajeid/1/4/5

10. Sahu PC, Inamdar IF, Sahu AC, ... Health seeking behaviour about gynaecological morbidities among ever married women of reproductive age group in a city of Maharashtra, India. Medica Innov [Internet]. 2022; Available from: https://search.ebscohost.com/login.aspx?direct=true&profile=ehost&scope=site&authtype=crawler&jrnl=22787526&AN=159202422&h=03q9a%2FQe7%2FqgKY4gqZgrBJ7CxzJWPVviWCHT1d15cUNj3xRKml8t0KmvxLO04FOrPM6Oy%2FTPWk5fnXFwsv34%2BA%3D%3D&crl=c

11. Ganganahalli PS, Singh CB. Factors Influencing Non-utilization of Antenatal Care Services from Government Sector among Rural Pregnant Women - A Hospital-based, Cross-sectional Study in Vijayapura District of North Karnataka. J Indian Med Assoc [Internet]. 2022;120(3):12–5. Available from: https://www.scopus.com/inward/record.uri?eid=2-s2.0-85130060821&partnerID=40&md5=7e747ee095b85a1bad23fc721bd39056

12. Sharma D, NK G, MM T. Prevalence of reproductive tract infection symptoms and treatment-seeking behavior among women: A community-based study. Indian J Sex Transm Dis AIDS [Internet]. 2018;39(2):79–83. Available from: https://pubmed.ncbi.nlm.nih.gov/30623176/

13. Mathew L, Francis LAJ. Prevalence of gynaecological morbidity and treatment seeking behaviour among married women in rural Karnataka: A cross sectional survey. J Krishna Inst Med Sci Univ [Internet]. 2017;6(3):84–93. Available from: https://www.scopus.com/inward/record.uri?eid=2-s2.0-85021775839&partnerID=40&md5=f7734dd7e17a1512744500798c124c09

14. Shastri DVD, Ram F. Health Seeking Behaviour and Utilisation of Reproductive Health Services for Gynaecological Problems--A Study Among Rural Women in Central India. Available SSRN 2579279. 2015;

15. Shingade PP, Kazi Y, ... Treatment seeking behavior for sexually transmitted infections/reproductive tract infections among married women in urban slums of Mumbai, India. South east asia journal …. pdfs.semanticscholar.org; 2015.

16. Chellan R. Socio-demographic determinants of reproductive tract infection and treatment seeking behaviour in rural Indian women. New Delhi: Centre for the Study of Regional …. 2004.

17. Shastri, Vishal and Ram F. Shastri, Vishal and Ram, Faujdar, Health Seeking Behaviour and Utilisation of Reproductive Health Services for Gynaecological Problems -- A Study Among Rural Women in Central India (March 16, 2015). Available at SSRN: https://ssrn.com/abstract=2579279 or . SSRN.

18. Surya B, Shivasakthimani R, Muthathal S, Prakash B, Loganathan S, Ravivarman G. A cross-sectional study on health-seeking behavior in relation to reproductive tract infection among ever-married rural women in Kancheepuram district, Tamil Nadu. J Fam Med Prim care [Internet]. 2021;10(9):3424–8. Available from: https://pubmed.ncbi.nlm.nih.gov/34760768/

19. Singh K, Kanika G. Factors affecting maternal health care seeking behaviour in northeast states, India: evidence from district level household survey-4 (2012-2013). Int J Res Med Sci. 2016.

20. Mathew L, Francis LAJ. Perceived barriers for utilization of health care system among married women with gynaecological morbidity in Udupi taluk, Karnataka. Indian J Public Heal Res Dev [Internet]. 2018;9(1):85–8. Available from: https://www.scopus.com/inward/record.uri?eid=2-s2.0-85041719564&doi=10.5958%2F0976-5506.2018.00016.5&partnerID=40&md5=fd23c2022474dab5f8a6da9625dcd3b4

21. MA K, BK P, Panigrahi P, KK B. Frequency and determinants of health care utilization for symptomatic reproductive tract infections in rural Indian women: A cross-sectional study. PLoS One [Internet]. 2019;14(12):e0225687. Available from: https://pubmed.ncbi.nlm.nih.gov/31805087/
